# SLC25A48 controls mitochondrial choline import and metabolism

**DOI:** 10.1016/j.cmet.2024.07.010

**Published:** 2024-08-06

**Authors:** Anthony R.P. Verkerke, Xu Shi, Mark Li, Yusuke Higuchi, Tadashi Yamamuro, Daisuke Katoh, Hiroshi Nishida, Christopher Auger, Ichitaro Abe, Robert E. Gerszten, Shingo Kajimura

**Affiliations:** 1 Division of Endocrinology, Diabetes and Metabolism, Beth Israel Deaconess Medical Center and Harvard Medical School, and Howard Hughes Medical Institute, Boston, MA, USA; 2 Division of Cardiovascular Medicine, Beth Israel Deaconess Medical Center and Harvard Medical School, Boston, MA, USA; 3 Department of Cardiology and Clinical Examination, Oita University, Faculty of Medicine, Oita, Japan; 4 Lead contact

## Abstract

Choline is an essential nutrient for the biosynthesis of phospholipids, neurotransmitters, and one-carbon metabolism with a critical step being its import into mitochondria. However, the underlying mechanisms and biological significance remain poorly understood. Here, we report that SLC25A48, a previously uncharacterized mitochondrial inner-membrane carrier protein, controls mitochondrial choline transport and the synthesis of choline-derived methyl donors. We found that SLC25A48 was required for brown fat thermogenesis, mitochondrial respiration, and mitochondrial membrane integrity. Choline uptake into the mitochondrial matrix via SLC25A48 facilitated the synthesis of betaine and purine nucleotides, whereas loss of SLC25A48 resulted in increased production of mitochondrial reactive oxygen species and imbalanced mitochondrial lipids. Notably, human cells carrying a single nucleotide polymorphism on the SLC25A48 gene and cancer cells lacking SLC25A48 exhibited decreased mitochondrial choline import, increased oxidative stress, and impaired cell proliferation. Together, this study demonstrates that SLC25A48 regulates mitochondrial choline catabolism, bioenergetics, and cell survival.

## In brief

Choline metabolism depends on its mitochondrial import, but the mechanism remains unknown. Verkerke et al. show that SLC25A48 controls mitochondrial choline transport and the synthesis of downstream metabolites, including betaine and purine nucleotides. Mitochondrial choline import via SLC25A48 is required for mitochondrial respiration, brown fat thermogenesis, and cell survival.

## Graphical Abstract

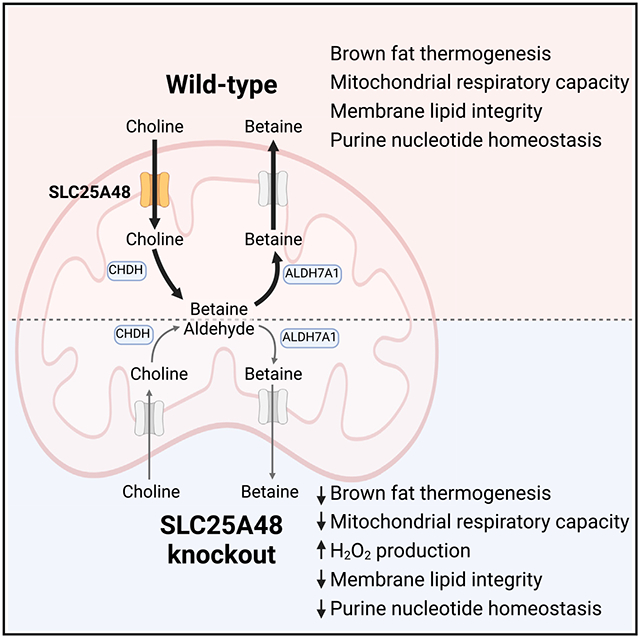

## INTRODUCTION

While the apparent role of mitochondria is to produce adenosine triphosphate (ATP), mitochondria also play a crucial role in catabolism, synthesis, and regulation of various metabolic processes through compartmentalized metabolism. Through these processes, mitochondria produce essential metabolites and signaling molecules that immensely impact cell-type selective functions in response to their metabolic needs.^1,2^ The import and export of mitochondria-compartmentalized metabolites is tightly regulated by the inner mitochondrial membrane (IMM). The IMM is impermeable to metabolites, and thus, the transport across the IMM relies on designated membrane-bound carrier proteins. Among them, solute carrier family 25 A (SLC25A) proteins belong to the largest family of carrier proteins that are mostly localized to the IMM.^3,4^ However, many of these proteins are considered “orphan” carriers as the specific substrates and biological functions remain uncharacterized.

Choline is one of the metabolites whose catabolism depends on mitochondrial import. It is a key nutrient that contributes to the synthesis of phospholipids and neurotransmitters and is also a major one-carbon donor within the cell.^5^ The conversion of choline to its one-carbon donor betaine (trimethylglycine) occurs within the mitochondria where a mitochondria-localized enzyme choline dehydrogenase (CHDH) converts choline to the intermediate betaine aldehyde, which is then converted to betaine by aldehyde dehydrogenase 7 family member A1 (ALDH7A1).^6^ Mitochondrial betaine is exported to the cytosolic compartment, where it serves as a methyl donor for the synthesis of methionine. The dynamic exchange of choline and betaine across the mitochondrial membrane is mediated by the IMM-bound carrier proteins, although the molecular identity of which remains unknown. In this regard, brown adipose tissue (BAT) serves as a useful discovery platform because of its highly enriched mitochondria, where many, if not all, IMM proteins are abundantly expressed. Besides, brown adipocytes exhibit unique features, including dense cristae structure, mitochondrial uncoupling respiration, and thermogenesis, which are useful indicators for determining the biological functions of uncharacterized IMM proteins.^7^ By employing cellular tools combined with metabolomics and *in vivo* physiology, this study aimed to characterize the previously unknown mitochondrial choline carrier.

## RESULTS

### SLC25A48 is required for BAT thermogenesis and mitochondrial membrane integrity

**Figure 1.**
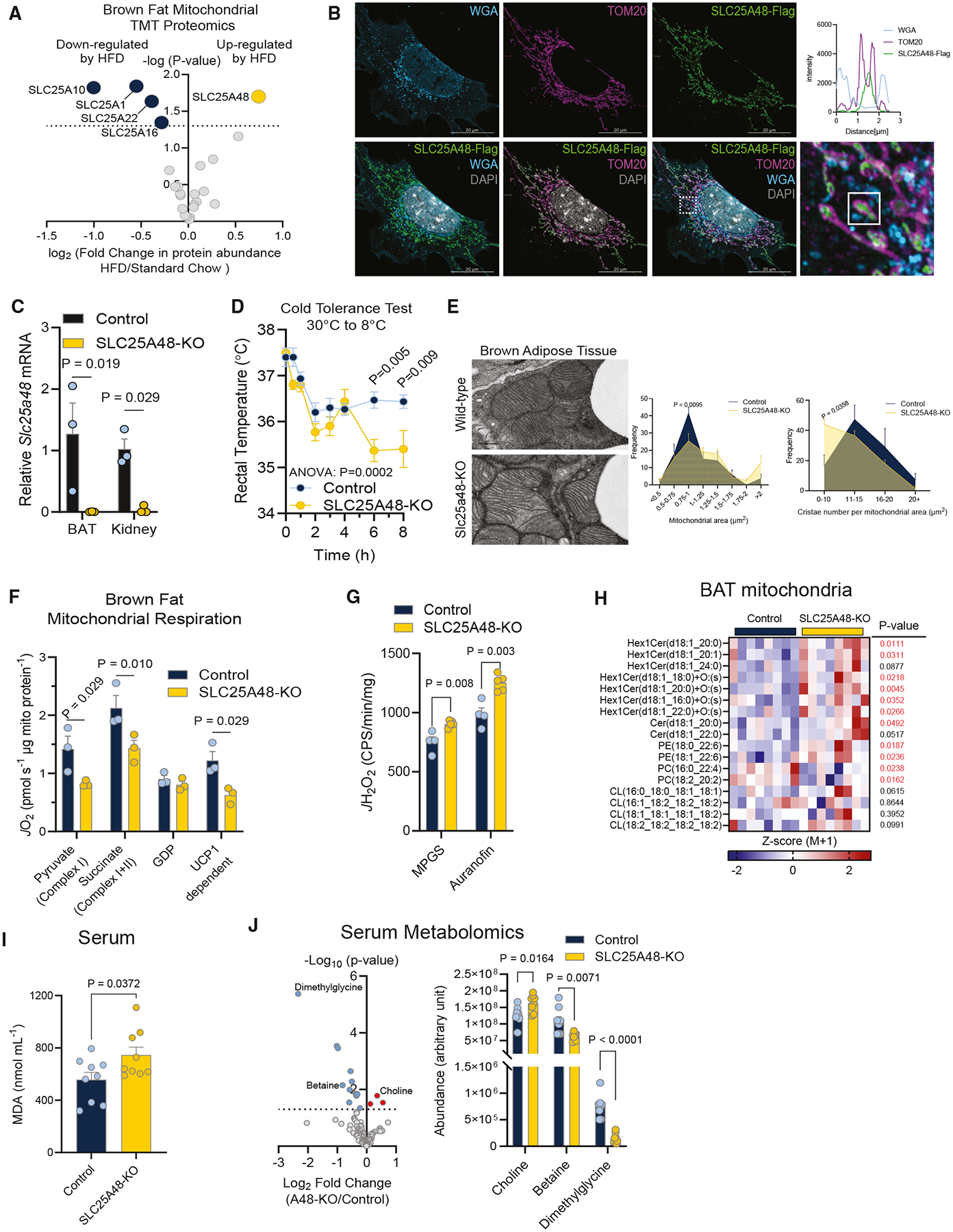
SLC25A48 is required for BAT thermogenesis and mitochondrial membrane integrity *in vivo* (A) SLC25A family protein abundance from BAT mitochondria of wild-type male mice fed a standard diet or high-fat diet for 8 weeks. *n* = 5 per group. (B) Representative immunofluorescent image of brown adipocytes expressing SLC25A48-FLAG. A plasma membrane marker (WGA), mitochondrial marker (TOM20), and nuclear marker (DAPI) were shown. Scale bar, 20 μm. (C) Relative SLC25A48 mRNA levels in indicated tissues from male control and SLC25A48-KO mice. *n* = 3 per group. (D) Rectal temperature of mice during cold tolerance test. *n* = 3 per group. (E) BAT mitochondrial structure of male control mice and SLC25A48-KO mice at 12 weeks old fed standard diet. Right: quantification of mitochondrial size and cristae density (*n* = 3 per group). Representative image. Scale bar, 0.5 μm. (F) BAT mitochondrial respiration. *n* = 3 per group. (G) H_2_O_2_ production in isolated BAT mitochondria from male control and SLC25A48-KO mice. *n* = 4 control, *n* = 5 SLC25A48-KO. (H) Lipidomic analysis of BAT mitochondria from male wild-type and SLC25A48-KO mice. *n* = 8 per group. (I) Serum levels of malondialdehyde (MDA) in male control and SLC25A48-KO mic on a standard diet. *n* = 9 per group. (J) Serum metabolomics from male control and SLC25A48-KO mice on a standard diet. *n* = 8 per group. Statistics: unpaired t test (A and G–J); two-way ANOVA with Holm-Šídák’s multiple comparisons test (C); two-way ANOVA with Šídák’s multiple comparisons test (D–F).

We started with quantitative tandem mass tag proteomics to identify mitochondrial proteins in the BAT of wild-type male mice fed a standard diet or high-fat diet (HFD, 60% kcal from fat) for 8 weeks. The analysis identified several mitochondrial proteins downregulated by HFD, including the citrate carrier (SLC25A1), the dicarboxylate acid carrier (SLC25A10), a glutamate carrier (SLC25A22), and a carrier associated with Graves’ disease (SLC25A16). In contrast, SLC25A48 was the only SLC25A protein that was significantly upregulated in response to HFD (Figure 1A). SLC25A48 is highly expressed in BAT in addition to the liver and kidney, while its expression is nearly undetectable in the heart and soleus muscle even though these tissues also contain high levels of mitochondria (Figure S1A). To determine the cellular localization, we performed high-resolution microscopy in brown preadipocytes expressing SLC25A48 with FLAG-tag (SLC25A48-FLAG) and probed for a plasma membrane marker (wheat germ agglutinin [WGA]), an outer mitochondrial member (OMM) marker (TOM20), and SLC25A48 with anti-FLAG (Figure 1B). The signal for SLC25A48 did not overlap with the plasma membrane marker WGA but rather aligned with the mitochondrial marker TOM20. High-resolution co-localization analysis revealed that SLC25A48 did not co-localize with the OMM, but the signal was found between OMM membranes. We then performed a proteinase degradation assay in isolated mitochondria from brown adipocytes expressing a control empty vector or SLC25A48-FLAG. In proteinase K-treated mitochondria, OMM marker TOM20 was degraded, while IMM marker ATP synthase F1 subunit alpha (ATP5A) was not disrupted, representing that the IMM was intact. SLC25A48 expression was present in the mitochondrial fraction and was not degraded by proteinase K treatment (Figure S1B). These results suggest that SLC25A48 is localized to the IMM. Over the course of brown adipocyte differentiation, *Slc25a48* mRNA expression was highly induced (Figure S1C).

To understand the role of SLC25A48 *in vivo*, we acquired mice with a germline knockout (KO) of SLC25A48 that lacked exon 4 of *Slc25a48*, which encoded two of the six transmembrane domains (Figure S1D). Mice with heterozygous deletion of *Slc25a48* were crossed to generate littermate controls and mice with homozygous deletion of *Slc25a48* (SLC25A48-KO) (Figures 1C and S1E). Male and female mice lacking *Slc25a48* developed normally and did not have noticeable differences in body mass or fasting blood glucose compared with littermate controls (Figures S1F and S1G). However, SLC25A48-KO mice displayed significantly impaired cold tolerance compared with littermate controls (Figure 1D). Although the gross BAT morphology appeared indistinguishable between the genotypes (Figure S1H), electron microscopy analyses found that the mitochondrial ultrastructure of SLC25A48-KO BAT was enlarged and contained less dense cristae than control BAT (Figure 1E). We also found a defect in BAT thermogenesis as mitochondrial respiration of isolated BAT via complex I and II was significantly attenuated in male SLC25A48-KO mice relative to littermate controls (Figure 1F). In the presence of guanosine diphosphate (GDP), we did not observe differences in respiration rate, suggesting that UCP1-mediated thermogenesis was attenuated in the BAT of SLC25A48-KO mice. The result was consistent in female mice (Figure S1I). The impairment in BAT thermogenesis was not due to changes in UCP1 and mitochondrial OXPHOS proteins (Figures S1J and S1K). Rather, genetic loss of SLC25A48 resulted in impaired complex I and II activities, suggesting the possibility that SLC25A48 loss might cause electron leak in the mitochondrial membrane. To test this, we measured mitochondrial respiration isolated from the BAT with substrates that fed into complex I and II. To measure maximal H_2_O_2_ production (*J*H_2_O_2_), thioredoxin reductase was inhibited by auranofin. We found that mitochondrial H_2_O_2_ production (*J*H_2_O_2_) was significantly elevated in the BAT of SLC25A48-KO mice relative to that of control mice (Figure 1G).

Next, we examined if SLC25A48 controls the maintenance of mitochondrial lipid integrity for optimal electron flow. Lipidomics of BAT-derived mitochondria showed that SLC25A48 deletion resulted in elevated hexosylceramides (HexlCers) (Figure 1H), which are known to be associated with increased lipid peroxidation and ROS accumulation.^8–10^ Of note, several HexlCers species contained an oxygen adduct, suggesting elevated oxidative stress in SLC25A48-KO BAT mitochondria. SLC25A48-KO BAT also contained elevated ceramides, which are implicated in the production of mitochondrial ROS by inhibiting complex III and regulating nicotinamide adenine dinucleotide (NAD) phosphate oxidase.^11–14^ Additionally, the abundance of some phosphatidylcholine (PC) and phosphatidylethanolamine (PE), membrane structural phospholipids, was altered in SLC25A48-KO mitochondria. For instance, PE species with very-long-chain polyunsaturated fatty acids on the sn-2 position PE (18:0_22:6 and 18:1_22:6) were elevated in SLC25A48-KO mitochondria, whereas PC species (16:0_22:4 and 18:2_20:2) were decreased in SLC25A48-KO mice. These changes appeared selective to ceramides and PC/PE lipid species because cardiolipin, a well-known mitochondrial membrane lipid species, was not different in the BAT between the genotypes. Consistent with elevated mitochondrial H_2_O_2_ production, serum levels of malondialdehyde (MDA), a lipid peroxidation marker, were higher in SLC25A48-KO mice than in control mice (Figure 1I). These data suggest that SLC25A48 is required for optimal electron flow in the mitochondria, partly through imbalanced mitochondrial membrane lipid species.

Notably, we found that SLC25A48 regulates systemic choline metabolism. Serum choline levels were significantly higher in SLC25A48-KO mice than in control mice, whereas serum levels of betaine and dimethylglycine, both of which are choline-derived metabolites, were significantly lower in SLC25A48-KO mice than in control mice (Figure 1J). Betaine and methionine metabolism were the two top enriched metabolic pathways of all differentially abundant metabolites (Figure S1L).

### The cell-autonomous role of SLC25A48 in the regulation of choline metabolism

**Figure 2.**
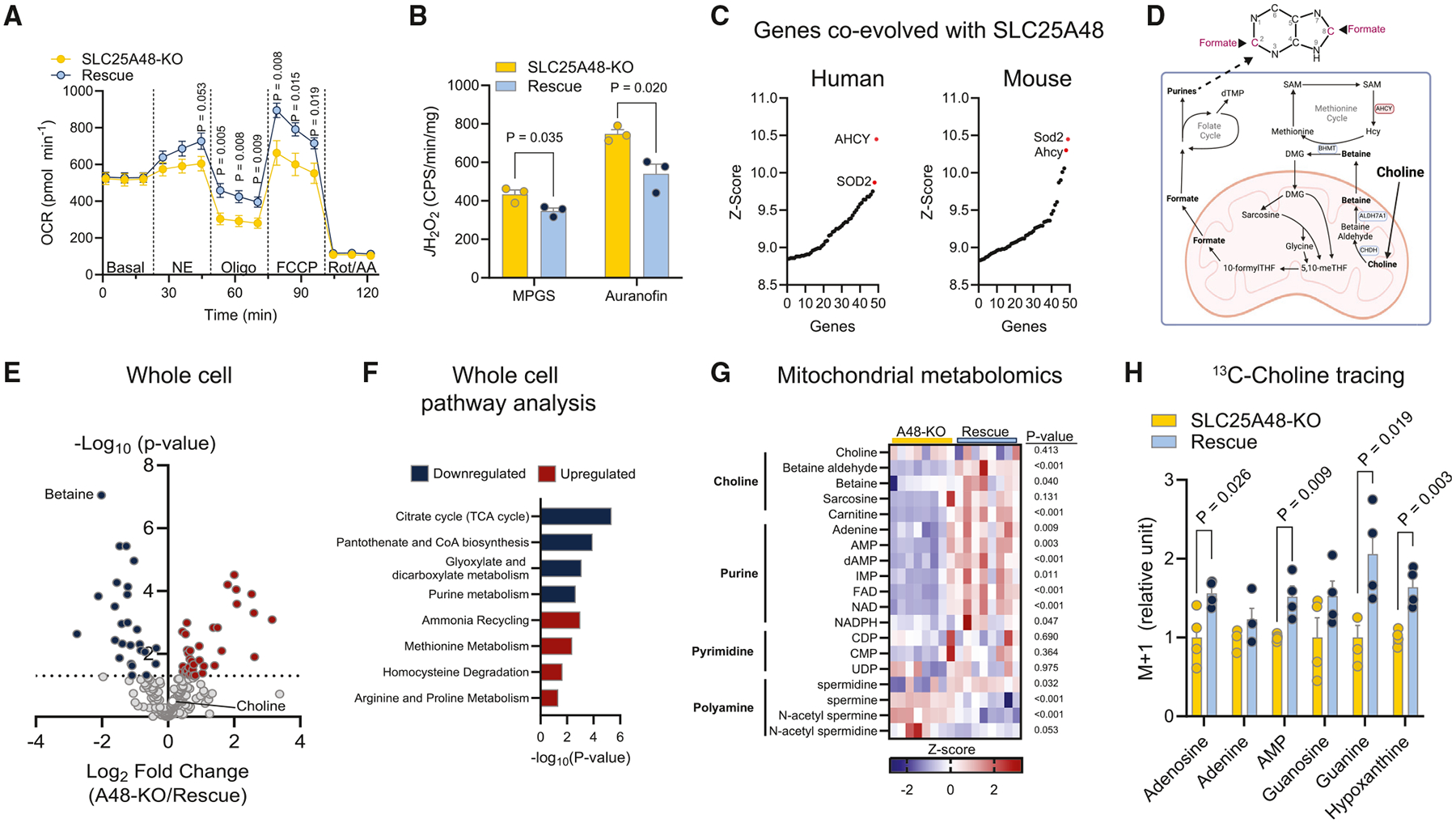
The cell-autonomous role of SLC25A48 in the regulation of choline metabolism (A) Cellular respiration of SLC25A48-KO and rescue (SLC25A48-KO^SLC25A48-FLAG^) brown adipocytes. *n* = 10 per group. (B) H_2_O_2_ production in isolated mitochondria of brown adipocytes. *n* = 3 per group. (C) Top 50 genes co-evolved with human *SLC25A48* (left) and mouse *Slc25a48* (right) across species ranked by *Z* score. (D) Schematic of mitochondrial contribution to one-carbon metabolites. Choline is imported into the mitochondrial matrix to be metabolized by CHDH and ALDH7A1 to form betaine. Methyl-groups from betaine are transferred to the methionine cycle or the folate cycle through production to formate. Formate is used at two positions (C2 and C8) in purine nucleotide synthesis. (E) Whole-cell metabolomics of SLC25A48-KO and SLC25A48-rescue brown adipocytes. *n* = 8 per group. (F) Pathway analysis of metabolites down- and upregulated in (E). (G) Mitochondrial metabolomics from brown adipocytes. *n* = 8 per group. Data represented as *Z* score. (H) ^13^C_2_-choline tracing to detect purine metabolites. SLC25A48-KO and rescue cells were incubated with ^13^C_2_-choline (1 mM) for 24 h. Values relative to SLC25A48-KO. *n* = 4 per group. Statistics: unpaired t test (A, B, E, G, and H).

We asked the extent to which a defect in BAT thermogenesis of SLC25A48-KO mice arises from the cell-autonomous function of brown adipocytes. To this end, we established stable brown adipocyte cells from the stromal vascular fraction of SLC25A48-KO BAT. Subsequently, we re-introduced a human SLC25A48-FLAG cDNA to SLC25A48-KO immortalized cells to establish SLC25A48-rescued cells (Figure S2A). We found no difference in brown adipogenesis or differentiation between SLC25A48-KO cells and SLC25A48-rescued cells (Figures S2B and S2C). However, norepinephrine-stimulated cellular respiration, in total and oligomycin-independent respiration, was significantly lower in SLC25A48-KO cells than in SLC25A48-rescued cells (Figure 2A). Carbonyl cyanide-p-trifluoromethoxyphenylhydrazone (FCCP)-induced maximum respiratory capacity was significantly lower in SLC25A48-KO cells relative to SLC25A48-rescued cells, although there was no difference in mitochondrial OXPHOS protein expression and extracellular acidification rate between the groups (Figures S2D and S2E). Importantly, mitochondrial H_2_O_2_ production in SLC25A48-KO brown adipocytes was significantly higher than in rescue cells both under basal and maximal conditions (Figure 2B). These results suggest the cell-autonomous function of SLC25A48 in cellular respiration.

Next, we employed phylogenic analysis based on the premise that proteins acting in the same pathways show similar patterns of sequence conservation across phylogenetic clades.^15^ Analyzing the similarity in sequence conservation patterns across a wide array of eukaryotic organisms may predict functional connections of a previously uncharacterized protein to known pathways. The phylogenic analysis of genes co-evolved with SLC25A48 across species revealed adenosylhomocysteinase (AHCY) as the top co-evolved gene in humans and second in mice, while superoxide dismutase 2 (SOD2) was the top co-evolved gene in mice and second in humans (Figure 2C). SOD2 is localized to the mitochondrial matrix and converts superoxide anions (O_2_^−^) to hydrogen peroxide (H_2_O_2_), which appears in agreement with the above results that SLC25A48 is involved in mitochondrial H_2_O_2_ production. AHCY is involved in the one-carbon methionine cycle and produces a reversible hydrolysis reaction, producing homocysteine and adenosine from s-adenosylhomocysteine (SAH).^16^ A gene ontology analysis of the top 25 co-evolved genes suggests that the one-carbon cycle pathway is highly enriched (Figure S2F). It is intriguing because one-carbon units in mammalian cells come from choline, serine, and glycine, all of which are imported into the mitochondria and metabolized to enter the folate cycle for the synthesis of purine nucleotides.^17^

While purine synthesis occurs in the cytosol, mitochondrial choline metabolism contributes to the synthesis of formate (Figure 2D). Specifically, the carbons on choline fall into the resulting backbone of the trimethyl-glycine product, which is released in the glycine cleavage system localized in the mitochondrial matrix. The glycine cleavage system breaks down glycine into CO_2_, ammonia, N^5^, and N^10^-methylene-tetrahydrofolate (5,10-meTHF). The carbon in 5,10-meTHF enters the folate cycle and provides a one-carbon unit in the form of formate that is used for the synthesis of purines at C2 and C8.^18^ To test whether SLC25A48 is involved in the one-carbon metabolic pathway, we performed liquid chromatography-mass spectrometry (LC-MS) metabolomics in whole cells as well as isolated mitochondria. Here, we employed the MITO-Tag expression system, which enabled us to perform rapid mitochondrial isolation and metabolite analyses.^19^ In whole cells, we observed reduced betaine in SLC25A48-KO cells as the most differentially abundant metabolite (Figure 2E). The pathway analysis of SLC25A48 downregulated metabolites suggests, in addition to purine metabolism, that TCA cycle, coenzyme A (CoA) biosynthesis, and glyoxylate and dicarboxylate metabolism were downregulated in SLC25A48-KO cells (Figures 2F and S2G). SLC25A48 upregulated metabolite pathway analysis also suggested SLC25A48 as a mediator of methionine metabolism and homocysteine degradation, both of which were implicated in choline metabolism. In isolated mitochondria, choline degradation metabolites, including betaine aldehyde and betaine, were significantly less in SLC25A48-KO cells than in SLC25A48-rescued cells (Figure 2G). SLC25A48-KO mitochondria contained significantly lower levels of purine nucleotides, such as adenine, adenosine monophosphate (AMP), dAMP, inosine monophosphate (IMP), and flavin adenine dinucleotide (FAD). Reduced fumarate and FAD levels may explain reduced mitochondrial complex II activity in SLC25A48-KO brown adipocytes. Likewise, mitochondrial contents of NAD and NAD phosphate (NADPH) were significantly lower in SLC25A48-KO cells compared with SLC25A48-rescued cells. In contrast, we did not find differences in pyrimidines, such as cytidine diphosphate (CDP), cytidine monophosphate (CMP), and uridine diphosphate (UDP).

Next, we determined the contribution of choline catabolism to purine synthesis by performing choline tracing using stable labeled choline (^13^C_2_-choline) to detect M + 1 label purine nucleotides by MS. We found that SLC25A48-KO cells contained significantly lower levels of choline-derived purine ^13^C-labeled nucleotides, including adenosine, AMP, and guanine, compared with SLC25A48-rescued cells (Figure 2H). These data suggest that one-carbon units from choline contribute to the synthesis of purine nucleotide pools, and SLC25A48 is involved in the process.

### SLC25A48 is required for mitochondrial choline import and catabolism

**Figure 3.**
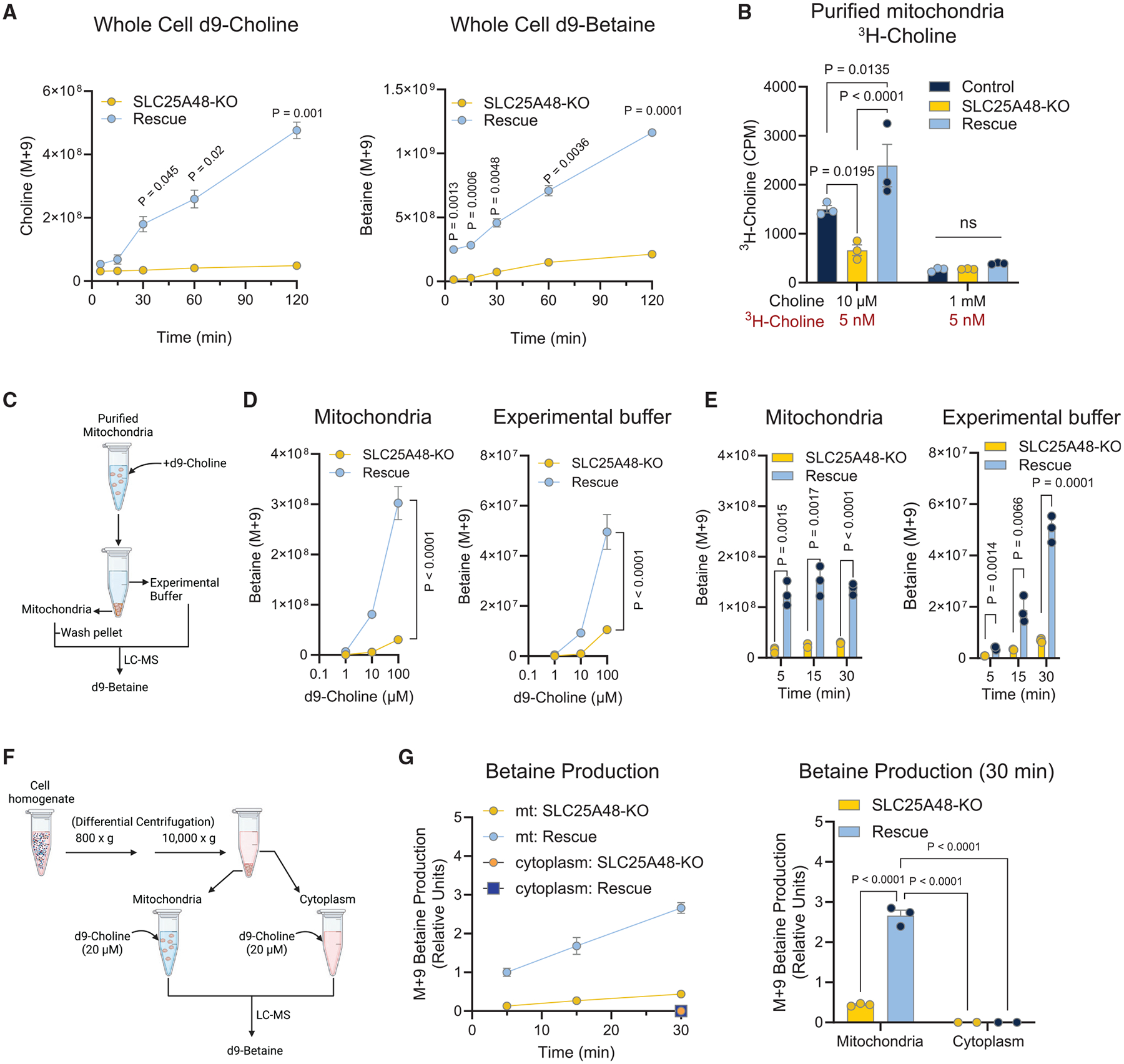
SLC25A48 is required for mitochondrial choline import and metabolism (A) Choline uptake into SLC25A48-KO and SLC25A48-rescued HEK293T cells. *n* = 4 per group. (B) Choline uptake in purified mitochondria by incubating with ^3^H-choline (5 nM) in the presence of non-labeled choline at 10 μM or 1 mM. *n* = 3 per group. (C) Schematic of d9-choline tracing experiment in (D) and (E). Isolated mitochondria from SLC25A48-KO and SLC25A48-rescue cells were incubated with d9-choline (M + 9) in a dose- or time-dependent manner. The mitochondria and the experimental buffer were separated through centrifugation and analyzed by LC-MS for d9-betaine (M + 9). (D) Dose-dependent betaine synthesis in mitochondria and experimental buffer. *n* = 3 per group per concentration. (E) Time-dependent betaine synthesis in mitochondria and experimental buffer with d9-choline at 20 μM. *n* = 3 per group per concentration. Statistic: unpaired t test. (F) Schematic of betaine production experiment in (G). Mitochondria were purified via differential centrifugation. The isolated mitochondria and cytoplasmic compartments were separately incubated with 20 μM d9-choline and analyzed by LC-MS to detect d9-betaine (M + 9). (G) Left: time course of betaine production from d9-choline in the isolated mitochondria and cytoplasmic compartments. Values relative to total M + 9 betaine at 5 min in SLC25A48 rescued mitochondria. Right: d9-betaine levels at 30 min of incubation. In the cytoplasmic compartment, d9-betaine only met the detection limit in 2 of 3 samples in both groups. *n* = 3 per group per time point. Statistics: two-way repeated-measures ANOVA with Šídák’s multiple comparisons test (A); two-way ANOVA with Tukey’s multiple comparisons test (B and G); two-way ANOVA with Šídák’s multiple comparisons test (D).

The above data led to the hypothesis that SLC25A48 regulates the production of choline-derived metabolites in the mitochondria. To test this, we generated stable cell lines in HEK293T cells with a KO of SLC25A48 and SLC25A48-rescued cells (Figure S3A). We confirmed that SLC25A48-rescued cells expressed SLC25A48 exclusively in the mitochondrial IMM (Figure S3B). Then, we incubated these cells with 20 μM of heavy-labeled choline (choline-d9) to assess cellular choline uptake and betaine production. We found that the production of choline-derived betaine (M + 9) was significantly higher in SLC25A48-rescued cells than controls within 5 min of incubation and thereafter, even though no statistical difference was seen in cellular choline uptake at the first 5–15 min of incubation (Figure 3A). At the later time points of incubation at 30 min and thereafter, we found a greater rate of choline uptake in SLC25A48-rescued cells than in controls.

Next, we incubated the isolated mitochondria with 5 nM of radiolabeled (^3^H) choline in the presence of a physiological level (10 μM) or excess amounts (1 mM) of unlabeled choline for 5 min, washed them, and assessed the radioactive activity in the mitochondria. We found that mitochondrial choline uptake was significantly lower in SLC25A48-KO mitochondria compared with wild-type controls with endogenous SLC25A48 expression, whereas SLC25A48 expression significantly increased mitochondrial choline uptake (Figure 3B). The ^3^H-choline signal was substantially decreased in all groups when co-incubated with excess (1 mM) unlabeled choline, validating the specificity of the signal for choline. We next incubated isolated mitochondria with heavy-labeled (d9)-choline at 1–100 μM. After 15 min of incubation, we washed off the labeled choline and detected d9-betaine (M + 9) in the mitochondrial pellet and experimental buffer (Figure 3C). SLC25A48-rescued mitochondria had significantly higher levels of d9-betaine both in the mitochondrial pellet and in the buffer compared with SLC25A48-KO (Figure 3D). The results suggest that choline is converted into betaine in the mitochondria and exported outside the mitochondria. A time course experiment with d9-choline (20 μM) showed that the choline-to-betaine conversion occurred within 5 min and continued to increase in a time-dependent manner (Figure 3E). To further determine if choline-derived betaine synthesis occurs within the mitochondria or also in the cytoplasmic compartment, we next isolated the mitochondrial fraction or the cytoplasmic fraction containing plasma membrane, Golgi, and endoplasmic reticulum among cytosolic proteins but lacked mitochondria. Subsequently, we incubated the isolated mitochondria or the cytoplasmic compartment with d9-choline to detect d9-betaine (Figure 3F). We found that d9-betaine was detected in the mitochondrial compartment but not in the cytoplasmic compartment (Figures 3G and 3H). These results showed that mitochondrial choline uptake contributes primarily to the synthesis of betaine within the mitochondria.

In contrast, the following experiment suggests that SLC25A48 is not involved in mitochondrial FAD import, although SLC25A48-KO mitochondria exhibited reduced levels of FAD. First, we treated SLC25A48-KO and rescued cells with ^13^C_4_,^15^N_2_-riboflavin (M + 6) for 4 h before performing metabolomics in whole-cell and mitochondrial fractions (Figure S3C). In whole-cell fraction, we found a trend of lower unlabeled (M + 0) FAD levels in SLC25A48-KO cells, whereas the labeled fraction (M + 6) was unchanged (Figure S3D). In the mitochondrial fraction, unlabeled FAD was also significantly less, while labeled FAD levels were not different between the two groups (Figure S3E). This suggests the lower abundance of mitochondrial FAD in the SLC25A48-KO cells is likely due to reduced synthesis of purine nucleotides rather than changes in the flux of riboflavin to FAD or the mitochondrial import of FAD.

### Mitochondrial choline catabolism via SLC25A48 regulates cell proliferation and survival

**Figure 4.**
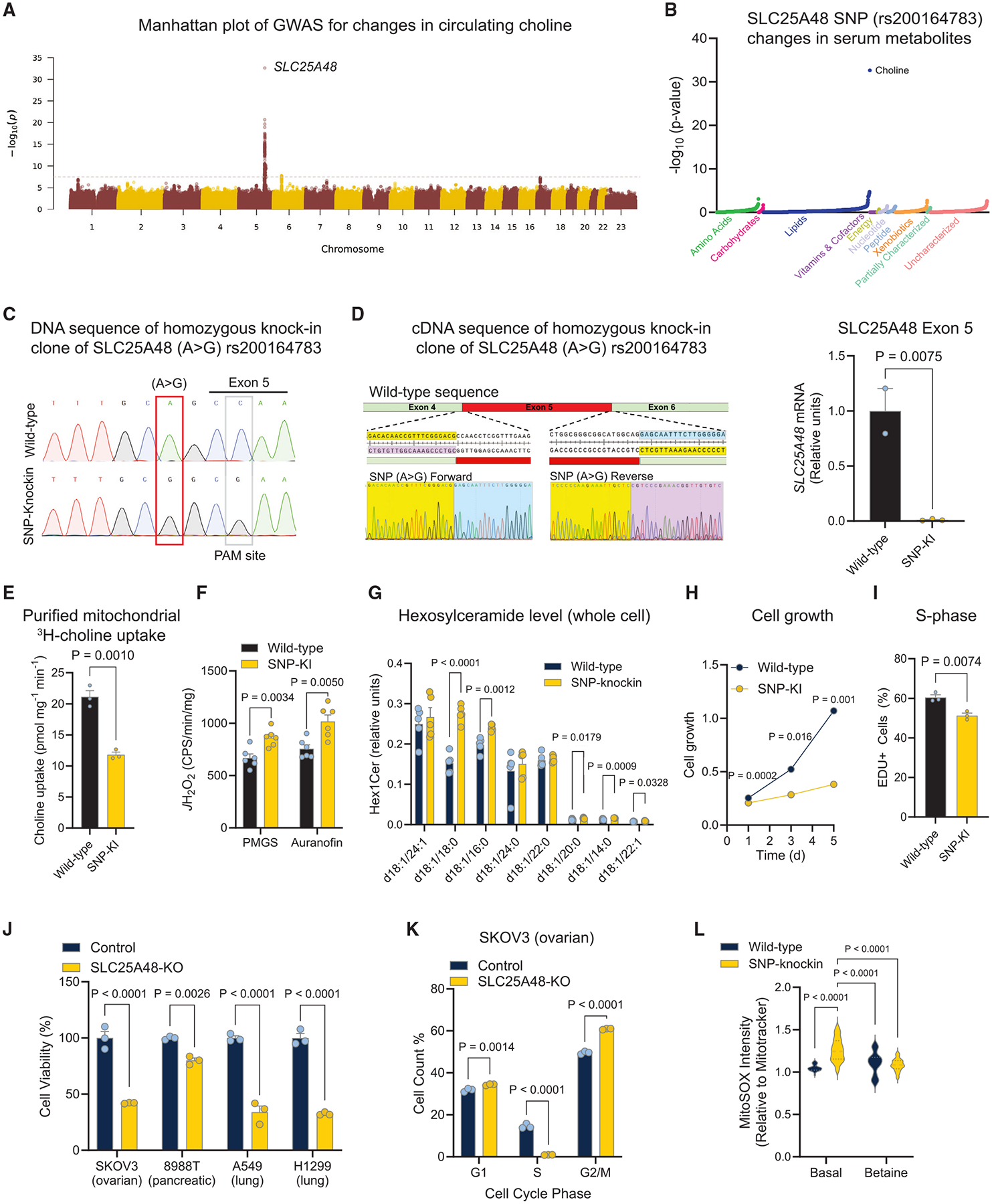
Mitochondrial choline catabolism via SLC25A48 regulates cell growth (A) Manhattan plot of GWAS for SNPs associated with circulating choline levels in 6,136 participants. (B) Association of *SLC25A48* SNP rs200164783 for changes in 1,391 metabolites. (C) DNA sequencing of wild-type control and homozygous knockin (SNP-KI) of *SLC25A48* SNP rs20016478 (A>G) in HEK293T cell. (D) cDNA sequencing from *SLC25A48* mRNA in SNP-KI cells. Right: mRNA expression of *SLC25A48* exon 5 region in wild-type control and SNP-KI cells by qPCR. *n* = 2 for wild-type and 3 for SNP-KI. (E) Choline uptake in the isolated mitochondria from wild-type control and SNP-KI cells. *n* = 3 per group. (F) H_2_O_2_ production in isolated mitochondria. *n* = 6 per group. (G) Indicated hexosylceramide levels. Values are normalized to the total hexosylceramide contents of control cells. *n* = 8 per group. (H) Cell growth of wild-type control and SNP-KI cells in DMEM containing 10% FBS. *n* = 3 per group. (I) Cell proliferation by EdU incorporation assays in (H). *n* = 3 per group. (J) Cell viability of indicated cancer cell lines by CRISPR-Cas9-mediated SLC25A48 deletion. *n* = 3 per group. (K) Cell-cycle analysis of SKOV3 ovarian cancer cells 16 h after transfection with control or SLC25A48-KO plasmid. *n* = 3 per group. (L) Mitochondrial H_2_O_2_ production in live cells in the presence or absence of betaine at 10 mM for 6 h. *n* = 32 for wild-type control cells for basal and betaine, 32 for SNP-KI basal, and 34 for betaine. Statistics: unpaired t test (D–G and I); two-way repeated-measures ANOVA with Šídák’s multiple comparisons test (H); two-way ANOVA with Šídák’s multiple comparisons test (J–L).

Genome-wide association studies (GWASs) with metabolomics in Finnish men from 6,136 participants^20^ provided new insights into the role of SLC25A48 in choline metabolism. The data found a strong correlation between plasma choline levels and the *SLC25A48* gene locus, with *SLC25A48* (rs200164783) being the most significant SNP (*p* = 2.3e–33) for choline across the genome (Figure 4A). The rs200164783 SNP was highly specific to changes in circulating choline of the 1,391 metabolites assessed (Figure 4B). This caught our attention because this SNP potentially alters a predicted splicing acceptor site located at the 3′ end of intron 4 of *SLC25A48* (AG>GG) that is evolutionarily conserved in mammals (Figure S4A).^21^ To test the functional consequence of this polymorphism, we then employed CRISPR-Cas9 genome editing technology with homology-directed repair (HDR) to introduce the *SLC25A48* (A>G) rs200164783 in human cells (HEK293 cells) (Figure S4B). In addition to the SNP (A>G) substitution, we mutated the PAM site to eliminate continuous Cas9 activity in transfected cells while keeping the identical amino acid coding sequence for alanine (GCC>GCG) of *SLC25A48*. Wild-type control cells were derived from the same parental cell populations as SNP knockin (SNP-KI) cells but lacked transfected genes, such as sgRNA and GFP. Subsequently, Sanger sequencing confirmed the A>G editing in these cells (Figure 4C). We found that SNP-KI cell-derived cDNA skipped exon 5 sequences on both forward and reverse strands. Consistently, qPCR primers spanning the exon 4–5 junction confirmed the deletion of exon 5 in SNP-KI cells but not in control cells (Figure 4D). This indicates that rs200164783 (A>G) causes a full exon 5 deletion of *SLC25A48*, which encodes for 2 transmembrane domains of the SLC25A48 protein (Figure S4C).

To examine the functional consequence of SLC25A48 SNP rs200164783 (A>G), we determined mitochondrial choline transport in SNP-KI cells and wild-type control HEK293T cells. We found that choline uptake into SNP-KI mitochondria was significantly blunted, which was equivalent to changes in SLC25A48-KO mitochondria (Figure 4E). Also, SNP-KI mitochondria generated significantly higher levels of H_2_O_2_ than wild-type cells under basal and maximally stimulated conditions (Figure 4F). Furthermore, lipidomic analysis identified changes in lipid species that resembled SLC25A48-KO BAT mitochondria. For example, levels of HexlCers, such as Hex1Cer (d18:1/d18:0), were significantly higher in SNP-KI cells than in wild-type control cells (Figure 4G). Together, these data suggest that cells carrying *SLC25A48* SNP rs200164783 (A>G) exhibited similar changes to SLC25A48-KO cells, including reduced mitochondrial choline uptake, elevated mitochondrial H_2_O_2_ production, and HexlCer accumulation.

These results motivated us to examine the extent to which *SLC25A48* SNP rs200164783 (A>G) affects cell growth. To this end, we performed cell growth assays in SNP-KI and wild-type control cells in full growth media (DMEM with 10% fetal bovine serum [FBS]) containing choline at a physiological concentration (28.5 μM). We found that SNP-KI cell growth was significantly reduced compared with wild-type controls (Figure 4H), although choline depletion blunted cell growth in wild-type control cells (Figure S4D). Furthermore, SNP-KI cells exhibited significantly lower EdU incorporation than control cells (Figure 4I). These results were consistent with the result that SLC25A48 deletion significantly attenuated cell proliferation, whereas ectopic expression of SLC25A48 in KO cells sufficiently rescued cell growth (Figure S4E).

Given the critical role of *de novo* purine nucleotide synthesis in cancer,^22^ we next asked if SLC25A48-mediated changes in cell proliferation extended to cancer cells. Here, we used four independent human cancer cells, ovarian cancer cells (SKOV3), pancreas adenocarcinoma (PA-TU-8988T), and non-small-cell lung cancer cell lines (A549 and H1299), that expressed SLC25A48. In all cancer cells tested, genetic loss of SLC25A48 by the CRISPR-Cas9 system resulted in a significant reduction in cell viability (Figures 4J and S4F). Within 24 h post induction of SLC25A48 depletion, we found over 50% of ovarian and lung cancer cells died, and 20% of pancreatic cancer cells were dead. In agreement with the finding in SNP-KI cells, ovarian cancer cells lacking SLC25A48 failed to initiate the G1-to-S phase transition (Figure 4K). Similarly, the depletion of SLC25A48 in U2OS osteosarcoma cells impaired the cell cycle in the transition from the G1-to-S phase (Figure S4G). Lastly, we asked to what extent exogenous betaine could rescue the cellular changes caused by SLC25A48 loss. We assessed mitochondrial ROS using MitoSOX in wild-type control and SNP-KI cells under standard growth media conditions (DMEM with 10% FBS but no betaine) or in the media supplemented with 10 mM betaine for 6 h. We found that mitochondrial ROS levels were significantly higher in SNP-KI cells than in control cells under the basal condition. Notably, 6 h of betaine supplementation significantly reduced mitochondrial ROS signal in SNP-KI cells to the equivalent levels in control cells (Figure 4L). However, betaine supplementation was insufficient to restore cell growth of SNP-KI cells (Figure S4H). The data suggest that pathways other than mitochondrial betaine synthesis are involved in the cell growth attenuation in SLC25A48-depleted cells.

## DISCUSSION

This study suggests that SLC25A48 mediates the import of choline into the mitochondrial matrix to allow for the conversion of choline to downstream metabolites, including betaine. Choline metabolism via SLC25A48 plays a key role in mitochondrial respiratory capacity, optimal electron flow, and the maintenance of mitochondrial membrane integrity. Intriguingly, the mitochondrial phenotype of SLC25A48-KO mice is similar to that of mice lacking CHDH, the first catabolic enzyme of choline in the mitochondrial matrix, which exhibits poor mitochondrial cristae density, low ATP levels, and reduced mitochondrial membrane potential in the sperm from *Chdh* KO mice.^23^ Furthermore, the loss of a plasma membrane choline transporter, feline leukemia virus subgroup C cellular receptor 1 (FLVCR1), resulted in enlarged mitochondria and reduced cristae density.^24^ We note that a plasma-membrane choline transporter-like protein 1 (CLT1, *SLC44A1*) was reported to mediate mitochondrial choline transport.^25^ However, MitoCarta 3.0^26^ found no detection of SLC44A1 in the mitochondria. Our study showed the requirement of SLC25A48 for mitochondrial choline import and catabolism. Nevertheless, labeled choline was still detected in the mitochondrial fraction of SLC25A48-KO cells, albeit to a substantially lesser extent. This suggests the possibility of redundant mitochondrial choline carriers in tissues, such as the heart and soleus, where SLC25A48 expression is low. The molecular identity of the additional choline transport pathway awaits future investigation.

The critical role of SLC25A48 for the synthesis of methyl-donor betaine immediately implicated the possible role in cell proliferation. The *de novo* synthesis of purine nucleotides requires one-carbon donor, and diet-derived choline makes up nearly 60% of methyl-donors consumed.^27^ Choline is a highly consumed metabolite across NCI-60 cell lines,^28^ although serine is the preferred source of one-carbon units^29^ and the preferred source of one-carbon donors depends on cell type.^17^ Regardless, we found that a defect in mitochondrial choline catabolism, by the deletion of SLC25A48 and the subsequent reduction in the purine nucleotide pool, resulted in impaired G1-to-S phase transition and cell death in multiple cancer cell lines. These results suggest that limiting mitochondrial choline catabolism provides a unique approach to regulating cell proliferation in some cancer cells. How the SLC25A48-mediated choline catabolic pathway is regulated in pathophysiology, including cancer and metabolic disorders, is an important area of future research.

### Limitations of the study

First, this study is limited to the analyses of whole-body SLC25A48-KO mice. To understand the pathophysiological role of SLC25A48 in a cell/tissue-selective manner, it is important to examine cell-type-specific KO mice. Second, while SLC25A48 loss in cancer cells resulted in impaired cell growth, supplementation with the downstream product betaine was insufficient to rescue cell growth. This suggests that SLC25A48 may regulate other cellular processes that require further study.

## STAR★METHODS

### RESOURCE AVAILABILITY

#### Lead contact

Further information and requests for resources and reagents should be directed to and will be fulfilled by the lead contact, Shingo Kajimura (skajimur@bidmc.harvard.edu).

#### Materials availability

Mouse strains and plasmids generated in this study are available upon request from the lead contact.

#### Data and code availability

- For proteomic datasets the MS raw data and analysis files were deposited with the ProteomeXchange Consortium (http://proteomecentral.proteomexchange.org).^32^ The dataset identifier for the mitochondrial proteome (Figure 1A) is PXD043992. Metabolomic dataset LC-MS raw data for mouse serum (Figure 1J) are uploaded to the Metabolomics Workbench (https://www.metabolomicsworkbench.org), with a project ID ST003264 and DOI: https://doi.org/10.21228/M8QV5X.
- Source data and uncropped western blots are available in Data S1.
- This paper does not report original code.
- Any additional information required to reanalyze the data reported in this work paper is available from the lead contact upon request.

### EXPERIMENTAL MODEL AND STUDY PARTICIPANT DETAILS

#### Mouse Strains and husbandry

All animal experiments were performed in compliance with approved protocols by the Institutional Animal Care and Use Committee at Beth Israel Deaconess Medical Center. All mice were housed were housed under a 12 h light – 12 h dark cycle. Room temperature mice were housed at 23°C in ventilated cages with an ACH of 25. Thermoneutral housed mice, were placed in an incubator set to 30°C. Mice were fed a standard diet (Lab Diet 5008) and had free access to food and water. Mice were fasted 4 h prior to metabolic measurements of fasting blood glucose and mitochondrial respiration. Slc25a48-knockout mice were acquired from the Knockout Mouse Phenotyping Program (KOMP) at Jackson Laboratory (Strain# 051066-JAX) and backcrossed to wild-type mice (Jackson Laboratory, Strain# 000664). Slc25a48-heterozygous knockout mice were used for breeding littermate controls. No sex-specific differences were observed between SLC25A48-KO mice. Male mice were used unless specifically stated.

#### Cell Culture

The stromal vascular fraction (SVF) from the brown adipose tissue of Slc25a48-KO mice were immortalized by expressing the SV40 large T antigen as previously described.^33^ The base media for adipocyte cell culture was DMEM (Gibco 11965092), containing 10% FBS and 1% penicillin/streptomycin (Gibco 15140). To induce differentiation to brown adipocytes, confluent preadipocytes were treated with an induction cocktail containing 0.5 mM isobutylmethylxanthine (Sigma I5879), 125 nM indomethacin (Sigma I7378), 2 μg/mL dexamethasone (Sigma D4902), 850 nM insulin (Sigma: I6634), and 1 nM T3 (Sigma T2877) for 2 days. After induction, cells were changed to a maintenance medium of base medium supplemented with 850 nM insulin and 1 nM T3. Cells were kept in a maintenance medium for 4–6 days to reach full differentiation. For HEK 293T and cancer cell culture (SKOV3, 8988T, A549, H1299, U2OS), cells were grown and maintained in DMEM (Gibco 11965092), containing 10% FBS and 1% penicillin/streptomycin (P/S) (Gibco 15140). Choline depletion studies used CMRL 1066, without choline chloride (USBiological life sciences, C5900-07) supplemented with glutamax 1%, 10% FBS, and 1% P/S.

### METHOD DETAILS

#### Plasmid and Virus production

Codon optimized SLC25A48 was cloned from Addgene plasmid #131995 and cloned into pMSCV-blasticidin (Addgene #75085) with Flag-tag on C-terminus. All constructs were confirmed by sequencing. pMXs-3XHA-EGFP-OMP25 was obtained for MITO-Tag experiments (Addgene #83356). CRISPR/Cas9 plasmids for control and SLC25A48-KO (Santa Cruz Biotechnology; sc-418922, sc-414730, sc-414730-HDR) were transfected with UltraCruz transfection reagent and plasmid transfection medium (Santa Cruz Biotechnology; sc-395739, sc-108062). For virus production HEK293T packaging cells were transfected with 10 μg of retroviral or lentiviral plasmid and the packaging constructs through calcium phosphate method. After 48 h, the viral supernatant was collected and filtered through a 0.45 μm filter. Immortalized preadipocytes were infected with viral supernatant supplemented with 10 μg ml^−1^ polybrene for 24 h. Stable cell lines were selected with puromycin (10 μg ml^−1^) or blasticidin (10 μg ml^−1^). Cells expressing MITO-Tag construct were sorted for equally low abundant EGFP-positive cells.^34^

#### Generation of SLC25A48 (A>G) rs200164783 knock-in cells

HEK 293T cells were transfected with HDR primer of SNP mutation (TTGCCACCTTCTCTCCCCTGTCTTTGCGGCGAACCTC GGTTTGAAGTCCAGGGCAGTG) and Px458 plasmid containing GFP, spCas9, and sgRNA (CCTGGACTTCAAACCGAGGT) targeting SLC25A48. Guide RNA candidates were screened using CRISPOR, CRISPRdirect, and Benchling to select for highest on target and lowest off target score. 24–48 hours after transfection, GFP-positive cells were sorted into single colonies with flow cytometry (BD FACSymphony S6 Cell Sorter). The plasmid Px458 is transiently expressed and cells were screened for loss of plasmid to ensure sgRNA and Cas9 absence prior to extracting DNA with QuickExtract DNA Extraction Solution 1.0 (LGC Biosearch Technologies). Single colony clone DNA were screened for HDR transfection through restriction enzyme digestion. Selected clones were then sequenced to confirm specific DNA mutations.

#### Codon-optimized SLC25A48-Flag cDNA sequence

Human SLC25A48 cDNA with codon optimization generated by the RESOLUTE Consortium^35^ was acquired from Addgene (#131995). A Flag-tag sequence was added to the C-terminal to allow for protein detection and SLC25A48-Flag cDNA was cloned into pMSCV-blasticidin (Addgene #75085). Sequence of SLC25A48-Flag:

GGCATCCCCACCTCGCACACTGTCTGACCTGCTGCTGGCAAGCATGGTGGCAGGAGTGGTGTCTGTGGGACTGGGAGGACCAGTGGACCTGATCAAGATCCGGCTGCAGATGCAGACACAGCCCTTTAGAGATGCCAACCTGGGCCTGAAGTCCAGGGCAGTGGCACCTGCAGAGCAGCCAGCATACCAGGGACCAGTGCACTGCATCACCACAATCGTGCGGAATGAGGGACTGGCAGGACTGTATAGGGGAGCAAGCGCCATGCTGCTGAGAGATGTGCCCGGCTACTGTCTGTATTTCATCCCTTACGTGTTCCTGTCTGAGTGGATCACCCCAGAGGCATGCACAGGACCAAGCCCATGTGCCGTGTGGCTGGCAGGAGGAATGGCAGGAGCCATCTCCTGGGGCACCGCCACACCTATGGACGTGGTGAAGTCTAGACTGCAGGCCGATGGCGTGTACCTGAACAAGTATAAGGGCGTGCTGGACTGCATCAGCCAGTCCTACCAGAAGGAGGGCCTGAAGGTGTTCTTTAGGGGCATCACCGTGAATGCCGTGCGCGGATTCCCTATGAGCGCCGCAATGTTTCTGGGCTATGAGCTGTCCCTGCAGGCAATCAGGGGCGATCACGCAGTGACATCCCCAGACTACAAGGATGACGATGATAAATAA.

#### Cell Proliferation and Viability

For the assessment of cell viability, we used the MTS assay kit (Abcam, ab197010) or Cell Counting Kit-8 (CCK-8) (Dojindo, CK04) according to the manufacturing protocol. We seeded an equal number of cells (2,000 – 5,000 cells) in 96-well and allowed cells 24 hours to adhere before assaying baseline. For the assay we added 1:10 (reagent:cell culture media) of MTS assay reagent or CCK-8 assay reagent in each well and measured the absorbance of 490 nm for MTS assay or 450 nm for CCK-8 assay. For cell viability, cell viability was measured 24 h post-transfection of control or targeting plasmid.

#### Cell Cycle

Hour 16 post-transfection control and SLC25A48-KO were incubated with 10 μM EdU (Click-iT Plus EdU Flow Cytometry Assay Kit, Thermo Fisher Scientific, USA) for 1 h in DMEM/F12 media. Subsequently, cells were fixed according to the manufacture’s instruction. FxCycle Violet Ready Flow Reagent (Thermo Fisher Scientific) was used to measure the total DNA content, following the manufacturer’s instructions. Cell population (%) was calculated as the frequency of parent. All the cells were analyzed using a FACS Aria II equipped with 100 mm nozzle diameter and CytoFLEX. FlowJo software (version 10.8.1) and CytExpert (version 2.4.0.28) were used for data analyses. In HEK 293T cells, we used the EdU Staining Proliferation Kit (iFluor 488) (Abcam, ab219801) according to the manufacturer’s protocol with minor changes. After the incubation of 10 μM EdU, cells on the cover glass (Electron Microscopy Sciences, 72229-01) were fixed with 4%PFA (Santa Cruz, sc-281692) followed by the incubation of 0.1% Triton X-100 (Millipore Sigma, X100) for permeabilization. After washing with PBS, cells were incubated with EdU reaction buffer followed by the staining of DAPI (Fisher Scientific, 62247). EdU-positive cells were observed under a fluorescence microscope (ECHO Revolve microscope). We evaluated more than 200 cells per sample for quantification.

#### Cold Tolerance

Mice were acclimated to 30°C for 10 days prior to being single housed at 8°C for 8 hours. The rectal temperature of mice were monitored hourly using TH-5 thermometer (Physitemp).

#### Cellular Respiration

Oxygen consumption rate (OCR) and extracellular acidification rate in fully differentiated brown adipocytes was measured with the Seahorse XFe Extracellular Flux Analyzer (Agilent) in a 24-well plate. Experiment was run in seahorse assay media supplemented with 10 mM glucose, 2 mM glutamine, and 1 mM pyruvate. Cells were stimulated with 10 μM norepinephrine, 5 μM oligomycin, 5 μM carbonyl cyanide-p-trifluoromethoxyphenylhydrazone (FCCP), and 5 μM of both rotenone and antimycin A.

#### Electron Microscopy and Quantification

Tissues were immersion fixed in 2% glutaraldehyde (Electron Microscopy Sciences, Hatfield, PA) and 2.5% paraformaldehyde (Electron Microscopy Sciences, Hatfield, PA) in 0.1M Sodium Cacodylate (Sigma-Aldrich, Burlington, MA) pH 7.4 for at least 1hr at room temperature and then at 4°C overnight. Tissues were washed with 0.1M Sodium Cacodylate, and then post-fixed for 1hr at 4°C in 1% osmium tetroxide (Electron Microscopy Sciences) in 0.1M Sodium Cacodylate. Cells were then washed in DI water and incubated in 2% aqueous uranyl acetate (Electron Microscopy Sciences) overnight at 4°C. The following day, tissues were washed with DI water and then dehydrated at 4°C in a graded ethanol series. Tissues were then brought to room temperature and dehydrated with 100% ethanol (Sigma-Aldrich) followed by propylene oxide (Electron Microscopy Sciences). Infiltration with LX112 resin (Ladd Research Industries, Williston, VT), was followed by embedding in flat bottom Beem capsules (Electron Microscopy Sciences). The resulting blocks were sectioned using a Leica Ultracut E ultramicrotome (Leica Microsystems, Wetzlar, Germany) and sections placed on formvar and carbon-coated grids (Electron Microscopy Sciences). The sections were contrast stained with 2% uranyl acetate followed by lead citrate (Sigma-Aldrich), and imaged in a JEOL 1400 transmission electron microscope (JEOL, Peabody, MA) equipped with a Gatan Orius SC1000 digital CCD camera (Gatan, Pleasanton, CA). Mitochondrial ultrastructure was analyzed with ImageJ software. For mitochondrial area, the outer membrane was traced with free hand tool and area was normalized to scale. Mitochondrial cristae density was calculated by dividing the number of cristae per mitochondrial area.^24^

#### MITO-Tag mitochondrial isolation

Following established protocols,^34^ mitochondria were immunoprecipitated from brown adipocytes. In 100 mm cell culture dishes of differentiated brown adipocytes that expressed MITO-Tag construct (3xHA-EGFP -OMP25), cells were washed twice with PBS. Cells were then scraped with KPBS (136 mM KCl, 10 mM KH2PO4, pH 7.25). Cells were pelleted by 1000 × g for 2 min at 4°C. Whole cell fraction was extracted directly into 80% MeOH, and stored at −80°C for Cells were resuspended in 1 mL of KPBS. Mitochondrial fraction was homogenized with Teflon pestle and glass tube. Homogenized sample was centrifuged at 1000 × g for 2 min at 4°C. Supernatant was then added to magnetic anti-HAbeads (Pierce, 88836) and nutate for 4 min at 4°C. Next, beads were collected by 1 min on magnetic rack. Sample was aspirated and beads were washed 3 times with KPBS. After beads were washed, 80% MeOH was added to extract metabolites and samples were stored at −80C overnight. Samples were then vortexed for 1 min and then centrifuged at 20000 × g for 10 min at 4°C. Supernatant was then transferred to clean tube and dried via speed vac. Dried metabolites were stored at −80°C for up to 1 week until resuspension in LC/MS grade H_2_O for LC/MS analysis.

#### ^3^H-Choline Mitochondrial uptake

Cells were homogenized in mitochondrial isolation media (MIM) containing 300 mM sucrose, 10 mM HEPES, 1 mM EGTA. Homogenized samples were centrifuged at 800 × g for 5 min at 4°C and supernatant was transferred to a new tube and pelleted at 10000 × g for 10 min at 4°C. The 10000 × g pellet contains mitochondria. Mitochondrial pellets were resuspended in MIM and assessed for protein concentration. Uptake assays were performed using 100 – 200 μg of total mitochondrial protein in MIM buffer. To start assays, ^3^H-choline (5 nM, 0.04 μCi) was added to purified mitochondria in the presence of 10 μM or 1 mM unlabeled choline at room temperature (25°C) for 5 minutes. At 5 minutes of incubation, samples were centrifuged at 10,000 × g at 4°C for 10 min to separate mitochondria from the buffer containing ^3^H-choline. The experimental buffer was removed, and the mitochondrial pellet was washed with KPBS (136 mM KCl, 10 mM KH2PO4, pH 7.25). Washed mitochondrial pellets were then resuspended in KPBS and transferred to scintillation vial for counting.

#### Mitochondrial betaine production

Cells were homogenized in mitochondrial isolation media (MIM) containing 300 mM sucrose, 10 mM HEPES, 1 mM EGTA. Homogenized samples were centrifuged at 800 × g for 5 min at 4°C and supernatant was transferred to a new tube and pelleted at 10000 × g for 10 min at 4°C. The 10000 × g pellet contains mitochondria. Mitochondrial pellets were resuspended in MIM and assessed for protein concentration. Additionally, 10,000 × g supernatant was collected and assessed for protein concentration. Betaine production assays were performed using 200 μg of total mitochondrial protein or cytosolic protein in MIM buffer. To start assay d9-choline (1 – 100 μM) was added to samples at room temperature (25°C) and was incubated for 5 – 30 minutes. At respective time points, the mitochondrial samples were centrifuged at 10,000 × g at 4°C for 10 min to separate mitochondria from the experimental buffer. The experimental buffer was removed and mitochondrial pellets were washed with KPBS (136 mM KCl, 10 mM KH2PO4, pH 7.25). The washed mitochondrial pellet was stored at −80°C until metabolite extraction. Cytosolic samples were quelled by placing the tube on dry ice and samples were stored at −80°C until metabolite extraction.

#### Cellular d9-choline uptake

Cells were incubated with 100 μM d9-choline for 5 – 120 minutes in standard growth media (DMEM, 10% FBS, 1% P/S). After respective time, cells were washed with ice cold PBS, scraped, and assessed for total protein content as cell growth of 293T cells is significantly different. Equal protein was used for metabolite extraction.

#### Choline contribution to purine nucleotides

HEK 293T cells were incubated with 1 mM ^13^C_2_-choline for 24 hours in standard growth media (DMEM, 10% FBS, 1% P/S). After respective time, cells were washed with ice cold PBS, scraped, and assessed for total protein content as cell growth of 293T cells is significantly different. Equal protein was used for metabolite extraction.

#### Metabolomics

Labeled metabolomics was carried out with HRHA orbitrap instruments (Exploris 240/Orbitrap ID-X) coupled with Vanquish Horizon UHPLC system. Waters ACQUITY UPLC BEH Amide column at 25 °C (particle size, 1.7 μm; 100mm (length) × 2.1mm (i.d.)) was used for LC separation. Mobile phases A = 25mM NH_4_Ac and 25mM ammonium hydroxide in water, and B = 100% acetonitrile were used for LC separation. The linear gradient as follows: 95% B (0.0–1 min), 95% B to 65% B (1–7.0 min), 65% B to 40% B (7.0–8.0 min), 40% B (8.0–9.0 min), 40% B to 95% B (9.0–9.1 min), then stayed at 95% B for 5.9 min. The flow rate was 0.4 mL/min. The sample injection volume was 2 μL for cell lysate and 5 μL for media. ESI source parameters were set as follows: spray voltage, 3500 V or −2800 V, in positive or negative modes, respectively; vaporizer temperature, 350 °C; sheath gas, 50 arb; aux gas, 10 arb; ion transfer tube temperature, 325 °C. The full scan was set as: orbitrap resolution, 60,000; maximum injection time, 100 ms; scan range, 70–1050 Da. The ddMS2 scan was set as: orbitrap resolution, 30,000; maximum injection time, 60 ms; top N setting, 6; isolation width, 1.0 m/z; HCD collision energy (%), 30; Dynamic exclusion mode was set as auto. The labeling metabolomics was quantified by Compound Discoverer 3.3. For unlabeled metabolomics, water soluble polar metabolites were injected an analyzed on a 5500 QTRAP hybrid triple quad quadrupole mass spectrometer (AB/SCIEX) coupled to a Prominence UFLC HPLC system (Shimadzu) with selected reaction monitoring (SRM) with positive/negative polarity switching.^36^ Peak areas were integrated using Multi-Quant 2.1 software.

For serum metabolomics 10 μL serum was mixed with 90 μL extraction solvent of methanol: acetonitrile: water 40: 40: 20 at −20°C, and then vigorously vortexed. Frozen tissues were ground to power using Cryomill (Retsch) at liquid N2 temperature. The tissue was extracted using methanol: acetonitrile: water 40: 40: 20 at a ratio of 1 mg tissue / 40 μL solvent at −20°C. All samples were then conditioned to −4°C, and centrifuged at 16,000 rpm/min. The supernatant was transferred to LC-MS tubes for analysis.

Chromatographic separation was achieved using XBridge BEH Amide XP Column (2.5 μm, 2.1 mm × 150 mm) with guard column (2.5 μm, 2.1 mm × 5 mm) (Waters, Milford, MA). Mobile phase A was water: acetonitrile 95:5, and mobile phase B was water: acetonitrile 20:80, both phases containing 10 mM ammonium acetate and 10 mM ammonium hydroxide. The linear elution gradient was: 0 ~ 3 min, 100% B; 3.2 ~ 6.2 min, 90% B; 6.5 ~ 10.5 min, 80% B; 10.7 ~ 13.5 min, 70% B; 13.7 ~ 16 min, 45% B; and 16.5 ~ 22 min, 100% B, with flow rate of 0.3 mL/ min. The autosampler was at 4°C. The injection volume was 5 μL. Needle wash was applied between samples using methanol: acetonitrile: water at 40: 40: 20. The mass spectrometry used was Q Exactive HF (Thermo Fisher Scientific, San Jose, CA), and scanned from 70 to 1000 m/z with switching polarity. The resolution was 120,000. Metabolites were identified based on accurate mass and retention time using an in-house library, and the software used was EI-Maven (Elucidata, Cambridge, MA).

#### LC-MS Lipidomic Analyses

Lipids were extracted in butanol/methanol (1:1) with 5 mM ammonium formate with 1:20 internal standard (Avanti, 330707). Mitochondria from brown adipose tissue were extracted as described in method “Mitochondrial respiration” and 300 μg of total mitochondrial protein was used for each sample. For whole cell lipidomics, 5 million cells were used for extraction.

Lipid extracts were analyzed using a Dionex Ultimate 3000 RSLC system (Thermo Scientific) coupled with a QExactive mass spectrometer (Thermo Scientific, Waltham, MA, USA) mass spectrometer. Chromatographic separation was achieved on an ACQUITY UPLC CSH C18 column (130Å, 1.7 μm, 2.1 mm × 100 mm) with an ACQUITY UPLC CSH C18 VanGuard pre-column (130Å, 1.7 μm, 2.1 mm × 5 mm) (Waters, Milford, MA) with column temperature at 50°C. For the gradient, mobile phase A consisted of an acetonitrile-water mixture (6:4), and mobile phase B was a 2-propanol-acetonitrile mixture (9:1), both phases containing 10 mM ammonium formate and 0.1% formic acid. The linear elution gradient was: 0–3 min, 20% B; 3–7 min, 20–55% B; 7–15 min, 55–65% B; 15–21 min, 65–70% B; 21–24 min, 70–100% B; and 24–26 min, 100% B, 26–28 min, 100–20% B, 28–30 min, 20% B, with a flow rate of 0.35 mL/ min. The autosampler was at 4°C. The injection volume was 5 μL. Needle wash was applied between samples using a mixture of dichloromethane-isopropanol-acetonitrile (1:1:1).

ESI-MS analysis was performed in positive and negative ionization polarities using a combined full mass scan and data-dependent MS/MS (Top 10) (Full MS/dd-MS2) approach. The experimental conditions for full scanning were as follows: resolving power, 70,000; automatic gain control (AGC) target, 1 × 106; and maximum injection time (IT), 100 ms. The scan range of the instrument was set to m/z 100–1200 in both positive and negative ion modes. The experimental conditions for the data-dependent product ion scanning were as follows: resolving power, 17,500; AGC target, 5 × 104; and maximum IT, 50 ms. The isolation width and stepped normalized collision energy (NCE) were set to 1.0 m/z, and 10, 20, and 40 eV. The intensity threshold of precursor ions for dd-MS2 analysis and the dynamic exclusion were set to 1.6 × 105 and 10 s. The ionization conditions in the positive mode were as follows: sheath gas flow rate, 50 arb; auxiliary (AUX) gas flow rate, 15 arb; sweep gas flow rate, 1 arb; ion spray voltage, 3.5 kV; AUX gas heater temperature, 325°C; capillary temperature, 350°C; and S-lens RF level, 55. The ionization conditions in the negative mode were as follows: sheath gas flow rate, 45 arb; auxiliary (AUX) gas flow rate, 10 arb; sweep gas flow rate, 1 arb; ion spray voltage, 2.5 kV; AUX gas heater temperature, 320°C; capillary temperature, 320°C; and S-lens RF level, 55.

Thermo Scientific LipidSearch software version 5.0 was used for lipid identification and quantitation. First, the product search mode was used during which lipids are identified based on the exact mass of the precursor ions and the mass spectra resulting from product ion scanning. The precursor and product tolerances were set to 10 and 10 ppm mass windows. The absolute intensity threshold of precursor ions and the relative intensity threshold of product ions were set to 30000 and 1%. Next, the search results from the individual positive or negative ion files from each sample were aligned within a retention time window (±0.25 min) and then all the data were merged for each annotated lipid with a retention time correction tolerance of 0.5 min. The annotated lipids were then filtered to reduce false positives by only including the lipids with a total grade of A or B.

#### Mitochondrial respiration and H_2_O_2_ emission

Brown adipose tissue was homogenized in buffer containing 300 mM sucrose, 10 mM HEPES, 1 mM EGTA, 1 mg/mL fatty acid-free BSA at pH 7.2. BAT homogenate was centrifuged at 600 × g for 5 min at 4°C and supernatant was centrifuged at 10000 × g for 10 min at 4°C to pellet mitochondria. Pelleted mitochondria were resuspended in homogenization buffer without BSA and normalized for equal protein through BCA assay. Mitochondrial respiration was measured with Oroboros O2k oxygraphs. For respiration experiments mitochondria were in buffer Z (100 mM MES potassium salt, 30 mM KCl, 10 mM KH_2_PO_4_, 5 mM MgCl_2_, 1 mM EGTA, 0.5 mg/ml fatty-acid free BSA pH 7.4). BAT mitochondria from male mice were stimulated with 0.5 mM malate, 5 mM pyruvate, and 10 mM succinate. BAT mitochondria from female mice were stimulated with 0.5 malate, 5 mM pyruvate, 5 mM glutamate, and 10 mM succinate. UCP1 was inhibited in both male and female mice with 4 mM GDP.

For mitochondrial H_2_O_2_ emission, mitochondria from brown adipose tissue, differentiated brown adipocytes, or HEK293T cells were extracted as described above. Protein concentration was determined by carrying out BCA assay. 50–70 μg of mitochondria were added to a cuvette containing buffer Z (2 mL), Amplex Red (10 μM), and horseradish peroxidase (3 U/mL). Signal was acquired following manufacturer’s instructions (Amplex UltraRed Reagent A36006, Invitrogen) at 37°C. Pyruvate (5 mM), malate (0.5 mM), glutamate (5 mM), succinate (20 mM), and auranofin (10 μM) were added as indicated in the main text. *J* H_2_O_2_ was determined as the rate of change over a specified period (100 seconds for each treatment).

#### Mitochondrial membrane localization

Cells were trypsinized and pelleted at 1100 × g for 2 min. Cell pellets were washed with phosphate buffer solution (PBS) and re-pelleted at 1100 × g for 2 min. Cell pellets were then homogenized in solution containing 22 mM mannitol, 75 mM sucrose, 1 mM EGTA, 30 mM Tris-HCl pH 7.4. Whole cell homogenate was split for whole cell fraction. Mitochondrial fraction was spun at 600 × g for 5 min and supernatant was pelleted for mitochondria at 7000 × g for 10 min. Assay was performed in 50 μL of 150 mM KCl, 10 mM HEPES, 200 μM CaCl_2_ buffer at pH 7.2. To mitochondrial fraction 2.5 μg of proteinase K was added and samples were incubated on ice for 10 min. After 10 min, Laemmli sample buffer was added and samples were boiled at 95°C for 10 min.

#### Proteomics

Isolated mitochondrial proteomes were reduced with 5 mM TCEP, alkylated with 10 mM Iodoacetamide, further reduced with 5 mM DTT, and precipitated using TCA (final concentration of 20%). The precipitated samples were washed three times with ice-cold acetone. Proteins were solubilized in a digestion buffer (1M urea, 200 mM EPPS pH 8.5) and digested with Lys-C overnight. The samples were further digested with trypsin for 6 hours at 37°C for six hours. The digested samples were labeled with TMTPro reagents (Thermo Fisher Scientific). Following incubation at room temperature for 2 hours, the reactions were quenched with hydroxylamine to a final concentration of 0.5% (v/v). Following TMT labeling, the samples were combined, and the pooled sample was de-salted using a Sep-pak. To perform mitochondrial TMT proteomics, labeled peptides were fractionated using Pierce High pH Reversed-Phase Peptide Fractionation Kit (Thermo Scientific). A total of 6 fractions were collected. Samples were subsequently acidified with 1% formic acid and vacuum centrifuged to near dryness. Each consolidated fraction was desalted by StageTip, and reconstituted in 5% acetonitrile, 5% formic acid for LC-MS/MS analysis. Data were collected on an Orbitrap Fusion Lumos Tribird mass spectrometer (Thermo Fisher Scientific) equipped with a Thermo Easy-nLC 1000 for online sample handling and peptide separations. The 100 μm capillary column was packed in-house with 35 cm of Accucore 150 resin (2.6 μm, 150Å; ThermoFisher Scientific). The peptides were separated using a 180 min linear gradient from 5% to 32% buffer B (90% ACN + 0.1% formic acid) equilibrated with buffer A (5% ACN + 0.1% formic acid) at a flow rate of 550 nL/min across the column. Data was collected using an SPS-MS3 method. The scan sequence for the Fusion Lumos Orbitrap began with an MS1 spectrum collected in the Orbirap (resolution - 120,000; scan range - 350 – 1,500 m/z; AGC target – 1,000,000; normalized AGC target – 250%; maximum ion injection time 50 ms; dynamic exclusion - 180 seconds). MS2 spectra were collected in the ion trap following collision-induced dissociation (AGC target – 15,000; normalized AGC target - 150%; NCE (normalized collision energy) – 35; isolation window - 0.5 Th; maximum injection time - 50ms). MS3 scans were collected in the Orbitrap following higher-energy collision dissociation (resolution – 50,000; AGC target – 100,000; normalized AGC target – 200%; collision energy – 55%; MS2 isolation window – 2; number of notches – 10; MS3 isolation window – 1.2; maximum ion injection time – 200 ms.

#### Proteomic Data Analyses

Database searching included all entries from the mouse UniProt Database (downloaded in May 2021). The database was concatenated with one composed of all protein sequences for that database in the reversed order.^37^ Raw files were converted to mzXML, and monoisotopic peaks were re-assigned using Monocle.^38^ Searches were performed with Comet^39^ using a 50-ppm precursor ion tolerance and fragment bin tolerance of 0.02. TMTpro labels on lysine residues and peptide N-termini +304.207 Da), as well as carbamidomethylation of cysteine residues (+57.021 Da) were set as static modifications, while oxidation of methionine residues (+15.995 Da) was set as a variable modification. Peptide-spectrum matches (PSMs) were adjusted to a 1% false discovery rate (FDR) using a linear discriminant after which proteins were assembled further to a final protein-level FDR of 1% analysis.^40^ TMT reporter ion intensities were measured using a 0.003 Da window around the theoretical m/z for each reporter ion. Proteins were quantified by summing reporter ion counts across all matching PSMs. More specifically, reporter ion intensities were adjusted to correct for the isotopic impurities of the different TMTpro reagents according to manufacturer specifications. Peptides were filtered to exclude those with a summed signal-to-noise (SN) < 160 across all TMT channels and < 0.5 precursor isolation specificity. The signal-to-noise (S/N) measurements of peptides assigned to each protein were summed (for a given protein).

#### Pathway enrichment

Gene ontology pathway enrichment^41,42^ was used to analyze biological processes from the top 25 genes from human SLC25A48 phylogene co-evolved prediction analysis.^15^ MetaboAnalyst 6.0 pathway analysis was employed to analyze whole cell metabolites less abundant in Slc25a48-KO compared to Slc25a48-rescued brown adipocytes.^43^

#### Immunofluorescent imaging

Cells were placed in a glass bottom dish (VWR 10810-054) and cultured for 24 hours. They were washed twice with PBS and fixed with 5% PFA at 37°C for 30 minutes. The cells were rinsed three times with PBS and permeabilized with 0.3% NP-40, 0.05% Triton X-100, and 0.1% bovine serum albumin (BSA) in PBS for 3 minutes. After three rinses with wash buffer (0.05% NP-40, 0.05% Triton X-100, and 0.2% BSA in PBS), the samples were blocked for 1 hour at room temperature with SuperBlock Blocking Buffer (Thermo Fisher, 37515). The samples were then incubated with primary antibodies in wash buffer overnight at 4°C, washed three times with wash buffer, and incubated with secondary antibodies for three hours at room temperature, followed by another three washes with wash buffer. The primary antibodies used targeted TOM20 (Proteintech, 11802-1-AP) and FLAG (Cell Signaling, 8146S). Secondary antibodies were conjugated with Alexa Fluor 488 (ab150117), Alexa Fluor 647 (Invitrogen A21245), and Wheat Germ Agglutinin (WGA), conjugated with Alexa Fluor 555 (Thermo Fisher, W32464) was used for confocal microscopy with a Zeiss LSM900 microscope with Airyscan. Images were processed using Zeiss Zen.

For mitotracker and mitoSOX experiments, HEK293 cells were placed in a glass bottom dish (VWR 10810-054) and cultured for 24 hours. For betaine supplementation, cells were plated on a glass bottom dish and cultured for 16 hours in DMEM supplemented with GlutaMAX and 10% FBS, followed by 6 hours of culture with 10 mM betaine in DMEM supplemented with GlutaMAX and 10% FBS. Cells were then rinsed with DMEM without phenol red and incubated with 1 μM MitoSox Red (Thermo Fisher, M36008) and 100 μM MitoTracker Green FM (Cell Signaling #9074) diluted in DMEM without phenol red for 15 minutes at 37°C. Imaging was conducted with a Zeiss LSM900 confocal microscope using the following excitation/emission filters: 488 nm laser line with a 500–550 nm emission filter (to detect MitoTracker Green) and a 488 nm laser line with a 570–620 nm emission filter (to detect MitoSox). For quantification, the region of interest (ROI) was determined by the MitoTracker signal in each cell, and the intensity of MitoTracker and MitoSox within the ROI was measured using ImageJ FIJI software. The intensity ratio (MitoSox/MitoTracker) was used to quantify mitochondrial ROS production.

#### Immunoblotting

Brown adipose tissue and brown adipocyte homogenates were probed for proteins of mitochondrial oxidative phosphorylation (Abcam, ab110413) and UCP1 (Sigma-Aldrich, U6382).

#### Lipid peroxidation

Lipid oxidative stress in serum (20 μL) was determined by measuring malondialdehyde (MDA) with a commercially available kit (Abcam, ab118970) utilizing the colorimetric assay.

#### Quantitative RT-PCR (qPCR)

Total RNA was isolated from cells or tissue using Trizol (Invitrogen) according to manufacturer instructions. RNA was reverse transcribed using iScript cDNA synthesis kit (Biorad). PCR reactions were performed with Applied Biosystems QuantStudio 6 Flex using Sybrgreen (Biorad). Assays were performed in duplicate, and all results were normalized to 18S ribosomal RNA or 36B4. Values are relative to the mean of the control group. Primers used are listed in Table S1.

### QUANTIFICATION AND STATISTICAL ANALYSIS

Statistical analyses were performed using GraphPad Prism v10. All data are represented at mean ± s.e.m. Unpaired t-tests were used for two-group comparisons. One-way ANOVA with Tukey’s multiple comparison test or two-way ANOVA with Šídák’s multiple comparison test were used for experiments with multiple comparisons. The statistical test used and sample numbers for each experiment are specified in the figure legends. P < 0.05 was considered to be significant.

## Supplementary Material

Sup Figures

source data S1

## Figures and Tables

**Table:** KEY RESOURCES TABLE

REAGENT or RESOURCE	SOURCE	IDENTIFIER
Antibodies		
UCP1	Sigma-Aldrich	Cat# U6382; RRID: AB_261838
Total Oxphos cocktail	Abcam	Cat# ab110413; RRID: AB_2629281
Flag	Sigma-Aldrich	Cat# F1804; RRID: AB_262044
ATP5A	Abcam	Cat# ab14748; RRID: AB_301447
TOM20	Proteintech	Cat# 11802-1-AP; RRID: AB_2207530
TOM20	Santa Cruz	Cat# sc-11415; RRID: AB_2207533
HA magnetic beads	Thermo Fisher Scientific	Cat# 88836
DAPI	Fisher Scientific	62247
FLAG	Cell Signaling	8146S
Wheat Germ Agglutinin (WGA), conjugated with Alexa Fluor 555	Thermo Fisher	W32464
Alexa Fluor 488	Abcam	ab150117; RRID: AB_2688012
Alexa Fluor 647	Invitrogen	A21245; RRID: AB_2535813
Goat anti-Mouse IgG (H+L) Secondary Antibody, HRP	Thermo Fisher Scientific	Cat# 31430; RRID: AB_10960845
Goat Anti-Rabbit IgG H&L (HRP)	abcam	Cat# ab6721; RRID: AB_955447
Chemicals, peptides, and recombinant proteins		
Indomethacin	Sigma-Aldrich	Cat# I7378
Insulin	Sigma-Aldrich	Cat# I6634
Isobutylmethylxanthine (IBMX)	Sigma-Aldrich	Cat# I5879
Dexamethasone	Sigma-Aldrich	Cat# D4902
3,3′,5-Triiodo-L-thyronine (T_3_)	Sigma-Aldrich	Cat# T2877
L-(−)-Norepinephrine(+)-bitartrate salt monohydrate	Sigma-Aldrich	Cat# A9512
DMEM	Gibco	Cat# 11965092
Fetal Bovine Serum	ATLANTA biologicals	Cat# S11550
Penicillin-Streptomycin	Gibco	Cat# 15140
Basticidin S HCl (10 mg/mL)	Gibco	Cat# A1113903
Puromycin Dihydrochloride	Thermo Fisher Scientific	Cat# A1113803
0.05% Trypsin	Corning	Cat# MT25052CI
cOmplete, EDTA-free Protease Inhibitor Cocktail	Roche	Cat# 11873580001
Phosphatase inhibitor cocktail 2	Sigma-Aldrich	Cat# P5726
Phosphatase inhibitor cocktail 3	Sigma-Aldrich	Cat# P0044
Dextrose	Sigma-Aldrich	Cat# D9434
Bovine Serum Albumin	Sigma-Aldrich	Cat# A1595
Laemmli sample buffer 4x	Bio-rad	Cat# M6773
Proteinase K	Thermo Fisher Scientific	Cat# 25530049
KCl	Sigma-Aldrich	Cat# P9541
HEPES	Sigma-Aldrich	Cat# H43375
UltraCruz transfection reagent	Santa Cruz Biotechnology	Cat# sc-395739
Plasmid transfection medium	Santa Cruz Biotechnology	Cat# sc-395739
Polybrene	Sigma-Aldrich	Cat# TR-1003-G
KH_2_PO_4_	Sigma-Aldrich	Cat# P5655
Malic Acid	Sigma-Aldrich	Cat# M6773
Pyruvate	Sigma-Aldrich	Cat# P4562
Glutamic acid potassium salt	Sigma-Aldrich	Cat# G1501
Succinic acid	Sigma-Aldrich	Cat# S9512
GDP	Sigma-Aldrich	Cat# G7127
Oligomycin	Cell Signaling Technology	Cat# 9996
Carbonyl cyanide-p-trifluoromethoxyphenylhydrazone (FCCP)	Sigma-Aldrich	Cat# C2920
Rotenone	Sigma-Aldrich	Cat# 557368
Antimycin A	Sigma-Aldrich	Cat# A8674
CaCl_2_	Sigma-Aldrich	Cat# C3306
Glucose	Sigma-Aldrich	Cat# G8270
MgCl_2_	Sigma-Aldrich	Cat# M2393
NaCl	Sigma-Aldrich	Cat# S7653
Na_2_HPO_4_	Fluka	Cat# 71639
NaH_2_PO_4_	Sigma-Aldrich	Cat# 71505
Tris	Sigma-Aldrich	Cat# 11814273001
EGTA	Sigma-Aldrich	Cat# E4378
n-dodecyl β-D-maltoside	Sigma-Aldrich	Cat# D4641
DTT	Sigma-Aldrich	Cat# 43816
Glycerol	Sigma-Aldrich	Cat# G7793
D9-Choline	Cambridge Isotope Laboratories	Cat# DLM-549-PK
3C4,15N2-Riboflavin	Cambridge Isotope Laboratories	Cat# CNLM-8851-PK
PBS	Gibco	Cat# 10010023
Phenylhydrazone	Sigma-Aldrich	Cat# C2920
Phosphate buffer solution	Thermo Fisher Scientific	Cat# P5244
Chloroform	Sigma-Aldrich	Cat# 650498
Acetonitrile	Thermo Fisher Scientific	Cat# A955
Methanol	Thermo Fisher Scientific	Cat# A456
D8-Phe	Cambridge Isotope Laboratories	Cat# DLM-372-1
D8-Val	Cambridge Isotope Laboratories	Cat# DLM-7784-PK
ammonium hydroxide	Thermo Fisher Scientific	Cat# 60-023-92
Water LC/MS	Thermo Fisher Scientific	Cat# W64
SDS	Thermo Fisher Scientific	Cat# AM9820
EDTA	Sigma-Aldrich	Cat# E5134
NP-40	Boston Bioproducts	Cat# P-877
Deoxycholate	Sigma-Aldrich	Cat# D6750
Triton-X 100	Thermo Fisher Scientific	Cat# BP151
Tween 20	Thermo Fisher Scientific	Cat# BP337
KOH	Boston Bioproducts	Cat# BZ-8038
BSA	Sigma-Aldrich	Cat# A7906
XF calibrant solution	Agilent	Cat# 100840-000
CMRL1066 w/L-Glutamine, w/o Choline Chloride, Sodium Acetate	USBiological life sciences	C5900-07
Glutamax	Thermo Fisher Scientific	35050061
Control Crispr/Cas9 plasmid	Santa Cruz Biotechnology	sc-418922
SLC25A48 Crispr/Cas9 KO plasmid	Santa Cruz Biotechnology	sc-414730
SLC25A48 HDR plasmid	Santa Cruz Biotechnology	sc-414730-HDR
QuickExtract DNA Extraction Solution 1.0	LGC Biosearch Technologies	QE0905T
TauI restriction enzyme	Thermo Fisher Scientific	ER1652
4%PFA	Santa Cruz Biotechnology	sc-281692
Circular cover glass	Electron Microscopy Sciences	72229-01
Choline chloride	Sigma Aldrich	C7527
Betaine	Sigma Aldrich	61962
3H-Choline	Revvity	NET109250
^13^C_2_-choline	Cambridge Isotope Laboratories	CLM-548-PK
Amplex Red	Invitrogen	A36006
Horseradish peroxidase (3 U/mL)	Thermo Fisher Scientific	31491
Auranofin	Sigma Aldrich	A6733
SuperBlock Blocking Buffer	Thermo Fisher Scientific	37515
Critical commercial assays		
Pierce BCA Protein Assay Kit	Thermo Fisher Scientific	Cat# 23225
Biorad gels 4–20%	Bio-rad	Cat# 4561096
Biorad gels 12% 10 well	Bio-rad	Cat# 4568044
iscript reverse transcription supermix for rt-qPCR	Bio-rad	Cat# 1708841
iTaq Universal SYBR Green Supermix	Bio-rad	Cat# 1725125
XFe24 FluxPak	Agilent	Cat# 102340-100
Glucometer	Abbott	Cat# Freestyle Lite
Glucose strips	Abbott	Cat# 70827
MTS Assay Kit	Abcam	Cat# ab197010
Click-iT Plus EdU Flow Cytometry Assay Kit	Thermo Fisher Scientific	Cat# C10632
FxCycle Violet Ready Flow Reagent	Thermo Fisher Scientific	Cat# R37166
EdU Staining Proliferation Kit (iFluor 488)	Abcam	ab219801
Malondialdehyde (MDA)	Abcam	Cat# ab118970
Glass bottom dish	VWR	10810-054
MitoSox Red	Thermo Fisher Scientific	M36008
MitoTracker Green FM	Cell Signaling	#9074
Deposited data		
BAT mitochondrial proteomics	Verkerke et al.^30^	PXD043992
Metabolomics	This paper	ST003264; DOI: https://doi.org/10.21228/M8QV5X
Experimental models: Cell lines		
Immortalized brown adipocytes	Yoneshiro et al.^31^	N/A
SLC25A48-FLAG immortalized brown adipocytes	This paper	N/A
SLC25A48-KO immortalized brown adipocytes	This paper	N/A
SLC25A48-KO^SLC25A48-Flag^ immortalized brown adipocytes	This paper	N/A
SLC25A48-KO 293T	This paper	N/A
SLC25A48-KO^SLC25A48-Flag^ 293T (Rescue)	This paper	N/A
SLC25A48 SNP-KI (rs200164783)	This paper	N/A
Experimental models: Organisms/strains		
Mouse: C57BL6J mice	Jackson Laboratory	Cat# 000664
Mouse: SLC25A48-knockout	Jackson Laboratory	Cat# 051066-JAX
Oligonucleotides		
A full list of qPCR primers in Table S1	This paper	N/A
Software and algorithms		
Biorender	Biorender	https://biorender.com/
Phylogene	Sadreyev et al.^15^	http://genetics.mgh.harvard.edu/phylogene/
Protter	Protter	https://wlab.ethz.ch/protter/start/
MetaboAnalyst Pathway Analysis	MetaboAnalyst	https://metaboanalyst.ca/
GO Enrichment Analysis	GeneOntology	http://geneontology.org/
GraphPad Prism 10	GraphPad Software	https://www.graphpad.com/scientific-software/prism/
Other		
Standard Diet	Lab Diet	Cat# 5008

## Highlights

- SLC25A48 is required for mitochondrial choline import and betaine synthesis
- Loss of SLC25A48 induces mitochondrial oxidative stress and respiratory impairment
- SLC25A48 controls choline contribution to purine nucleotide synthesis
- Cancer cells require mitochondrial choline metabolism via SLC25A48 for survival
