# Supplementary material for: SLC25A48 controls mitochondrial choline import and metabolism
Source: Cell Metab. Author manuscript; Available in PMC 2025 Mar 29. (PMC11953726; doi:10.1016/j.cmet.2024.07.010)
Supplement: Sup Figures [file NIHMS2063854-supplement-Sup_Figures.pdf]

Cell Metabolism, Volume *36*

Supplemental information

SLC25A48 controls mitochondrial choline import and metabolism

Anthony R.P. Verkerke, Xu Shi, Mark Li, Yusuke Higuchi, Tadashi Yamamuro, Daisuke Katoh, Hiroshi Nishida, Christopher Auger, Ichitaro Abe, Robert E. Gerszten, and Shingo Kajimura

Figure S1 (Related to Figure 1)

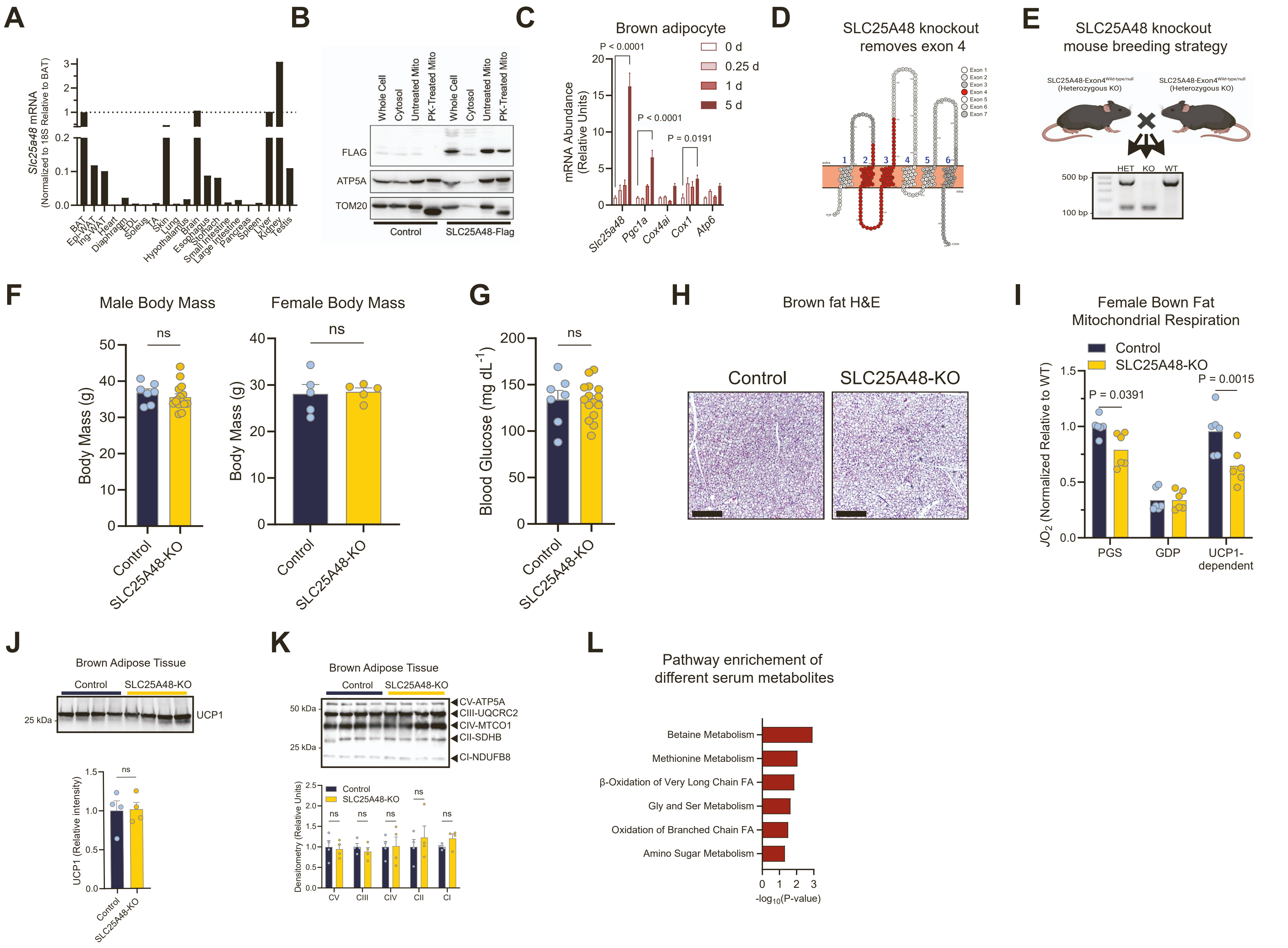

Figure S1. Analyses of SLC25A48 KO mice (Related to Figure 1)

A. Expression of SLC25A48 in 6-week-old wild-type mouse tissues. n= 1. BAT, brown adipose tissue; Epi-WAT, epididymal white adipose tissue; Ing-WAT, inguinal white adipose tissue; EDL, extensor digitorum longus; TA, tibialis anterior.

B. Proteinase K (PK) assay in brown adipocyte mitochondria. Mitochondria from brown adipocytes expressing control (empty-vector) or SLC25A48-Flag cDNA were exposed to a timed Proteinase K degradation to selectively degrade proteins on the outer mitochondrial membrane (OMM) and probed for OMM marker TOMM20, inner mitochondrial maker (IMM) ATP5A, and SLC25A48 through Flag. In control and SLC25A48-Flag the OMM marker TOMM20 was selectively degraded in PK treated mitochondria, while the IMM marker (ATP5A) was unchanged. Consistent with localization to the IMM, SLC25A48-Flag protein was not changed with PK treatment.

C. Expression of SLC25A48 and indicated differentiation marker genes during the course of mouse brown adipocyte differentiation. Brown adipocytes were collected at 0.25, 1, and 5 days after the addition of induction media (850 nM insulin, 1 nM T3, 125 nM indomethacin, 2 μg/mL dexamethasone, 0.5 mM isobutylmethylxanthine). After 2 days, cells were switched to maintenance media (850 nM insulin and 1 nM T3) and were maintained until full differentiation at day 5. n = 3 per time point. Statistic: two-way ANOVA with Tukey's multiple comparisons test. Bars represent mean and error shown as s.e.m.

D. The amino acid sequence of mouse SLC25A48. The Exon 4 contribution is highlighted. The strategy of SLC25A48-KO mouse was to delete Exon 4. Exon 4 is predicted to encode transmembrane domains 2 and 3 of SLC25A48.

E. Breeding strategy for SLC25A48-KO mice and littermate control mice. Mice with heterozygous deletion of SLC25A48 exon 4 were crossed to generate heterozygous knockout of SLC25A48 (HET), SLC25A48-KO (KO), or littermate controls. SLC25A48-KO mice and littermate wild-type control mice were studied.

F. Body mass of male and female control and SLC25A48-KO mice. n = 7 controls, 15 SLC25A48-KO male mice, 5 per group for females. Statistic: unpaired t-test. Bars represent mean and error shown as s.e.m. Individual values presented.

G. Fasting blood glucose in male control and SLC25A48-KO mice on a regular diet. Mice were fasted for 4 hours prior to measurement of glucose via handheld glucometer. n = 7 wild-type, 15 SLC25A48-KO. Statistic: unpaired t-test with individual values shown. Bars represent mean and error shown as s.e.m.

H. Representative images of Hematoxylin and eosin (H&E) staining of BAT from male control and SLC25A48-KO mice on a regular diet. Scale bar: 210 μm.

I. Brown adipose tissue isolated mitochondrial bioenergetics in female mice. Mitochondria were isolated from BAT of control and SLC25A48-KO mice and stimulated with pyruvate, glutamate, and succinate (PGS) to stimulate complex I and complex II respiration, followed by guanosine diphosphate (GDP) to inhibit UCP1. UCP1-dependent respiration was determined by subtracting GDP-respiration from complex I+II respiration (PGS). n = 6 per group. Statistic: two-way ANOVA with Šídák's multiple comparisons test. Bars represent mean and error shown as s.e.m., individual values presented.

J. Western blot for UCP1 in the BAT from male control and SLC25A48-KO mice. n = 4 per group. Statistic: unpaired t-test.

K. Western blot for indicated mitochondrial proteins in the BAT from male control and SLC25A48-KO mice. n = 4 per group. Statistic: unpaired t-test.

L. Pathway enrichment of differentially abundant serum metabolites (both up-regulated and down-regulated) from male control and SLC25A48-KO mice.

Figure S2 (Related to Figure 2)

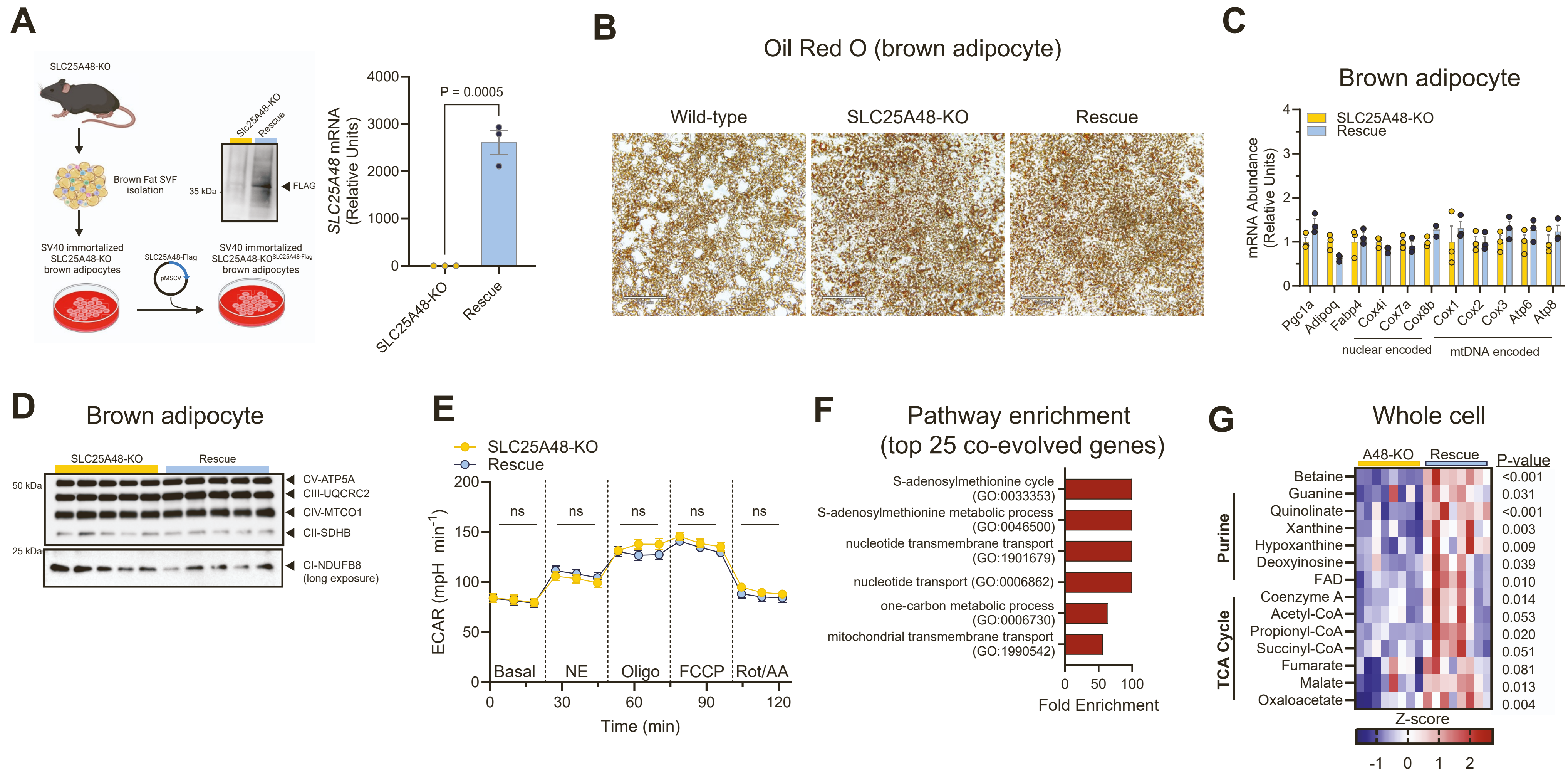

Figure S2. Cell-autonomous control of brown adipocytes by SLC25A48 (Related to Figure 2)

A. Schematic of developing SLC25A48-KO and SLC25A48-rescue brown adipocytes. The stromal vascular fraction (SVF) was isolated from the BAT of SLC25A48-KO mice and immortalized with SV40 through retroviral infection. Subsequently, human SLC25A48-Flag cDNA was expressed in SLC25A48-KO brown adipocytes by a retroviral vector. Protein expression of SLC25A48 in these cells was validated by immunoblotting for Flag. n = 3 per group. Statistic: unpaired t-test

B. Representative images of Oil-Red-O staining in SLC25A48-KO, rescue, and wild-type control brown adipocytes after 5 days of differentiation. Scale bar: 210 µm.

C. Relative mRNA levels of indicated brown adipocyte differentiation marker genes in (B). n = 3 per group. Statistic: two-way ANOVA with Šídák's multiple comparisons test.

D. Western blot for indicated mitochondrial complex proteins in brown adipocytes. Bands top to bottom complex IV (CV, ATP5A), complex III (CIII, UQCRC2), complex IV (MTCO1), complex II (SDHB), complex I (NDUFB8). n = 5 per group.

E. Extracellular acidification rate (ECAR) of SLC25A48-KO and SLC25A48-rescue brown adipocytes in response to norepinephrine (NE), oligomycin (oligo), carbonyl cyanide-p-trifluoromethoxyphenylhydrazone (FCCP), and rotenone and antimycin A (Rot/AA). n = 10 per group. Statistic is unpaired t-test. Circles represent mean and error shown as s.e.m.

F. Gene ontology pathway enrichment analysis for the top 25 genes to be co-evolved with SLC25A48 (Figure 2C).

G. Heatmap showing whole-cell metabolomics of SLC25A48-KO and SLC25A48-rescued brown adipocytes. Betaine was the most significantly less abundant metabolite in SLC25A48-KO brown adipocytes compared to SLC25A48-rescue brown adipocytes. n = 8 per group. Statistic: unpaired t-test. Data represented as z-score.

Figure S3 (Related to Figure 3)

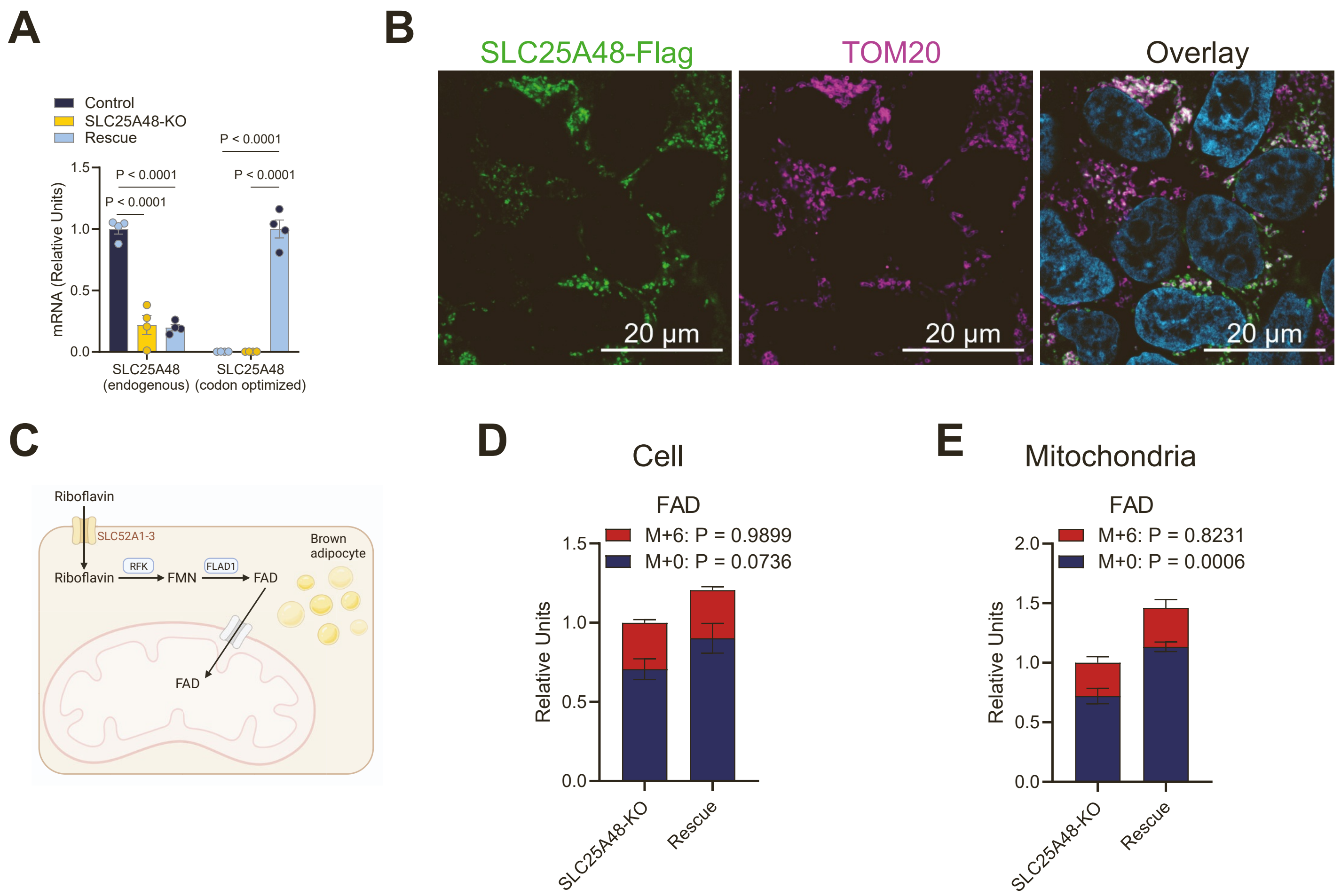

Figure S3. Mitochondrial metabolite uptake assays by SLC25A48 (Related to Figure 3)

A. Expression of endogenous *SLC25A48* and ectopically expressed *SLC25A48* (codon optimized) in HEK 293T control, SLC25A48-KO, and SLC25A48-rescued cells. Endogenous expression is relative to control and codon-optimized relative to Rescue. n = 4 per group. Statistic: two-way ANOVA with Tukey's multiple comparisons test. Bars represent mean and error shown as s.e.m., individual values presented.

B. Representative immunofluorescent imaging of SLC25A48-rescued HEK293T cells. Cells were labeled for SLC25A48 (Flag, green) and outer mitochondrial membrane maker (TOM20, magenta). Representative image. Scale bar: 20 µm.

C. Schematic of mitochondrial FAD transport tracing assay. Brown adipocytes were treated with labeled riboflavin ($^{13}C_4$,$^{15}N_2$ Riboflavin; M+6) for 4 hours. Riboflavin is imported into cells through plasma membrane transporters SLC52A1, SLC52A2, SLC52A3. In the cytosol, riboflavin is converted to flavin mononucleotide (FMN) by riboflavin kinase (RFK) and FMN is converted to flavin adenine dinucleotide (FAD) by flavin adenine dinucleotide synthetase (FLAD1). FAD import into the mitochondria is not fully understood. After 4 hours of treatment, cells were scraped and split into whole cell and mitochondrial fractions.

D. Whole-cell labeled (M+6, red) and unlabeled (D0, blue) FAD in SLC25A48-KO and SLC25A48-rescued brown adipocytes. n = 4 per group. Statistic: two-way ANOVA with Šídák's multiple comparisons test. Values relative to total FAD (M0 and M+6) of Slc25a448-KO. Bars represent mean and error shown as s.e.m.

E. Mitochondrial labeled (M+6, red) and unlabeled (D0, blue) FAD in SLC25A48-KO and SLC25A48-rescued brown adipocytes. n = 4 per group. Statistic: two-way ANOVA with Šídák's multiple comparisons test. Values relative to total FAD (M0 and M+6) of SLC25A48-KO. Bars represent mean and error shown as s.e.m.

Figure S4 (Related to Figure 4)

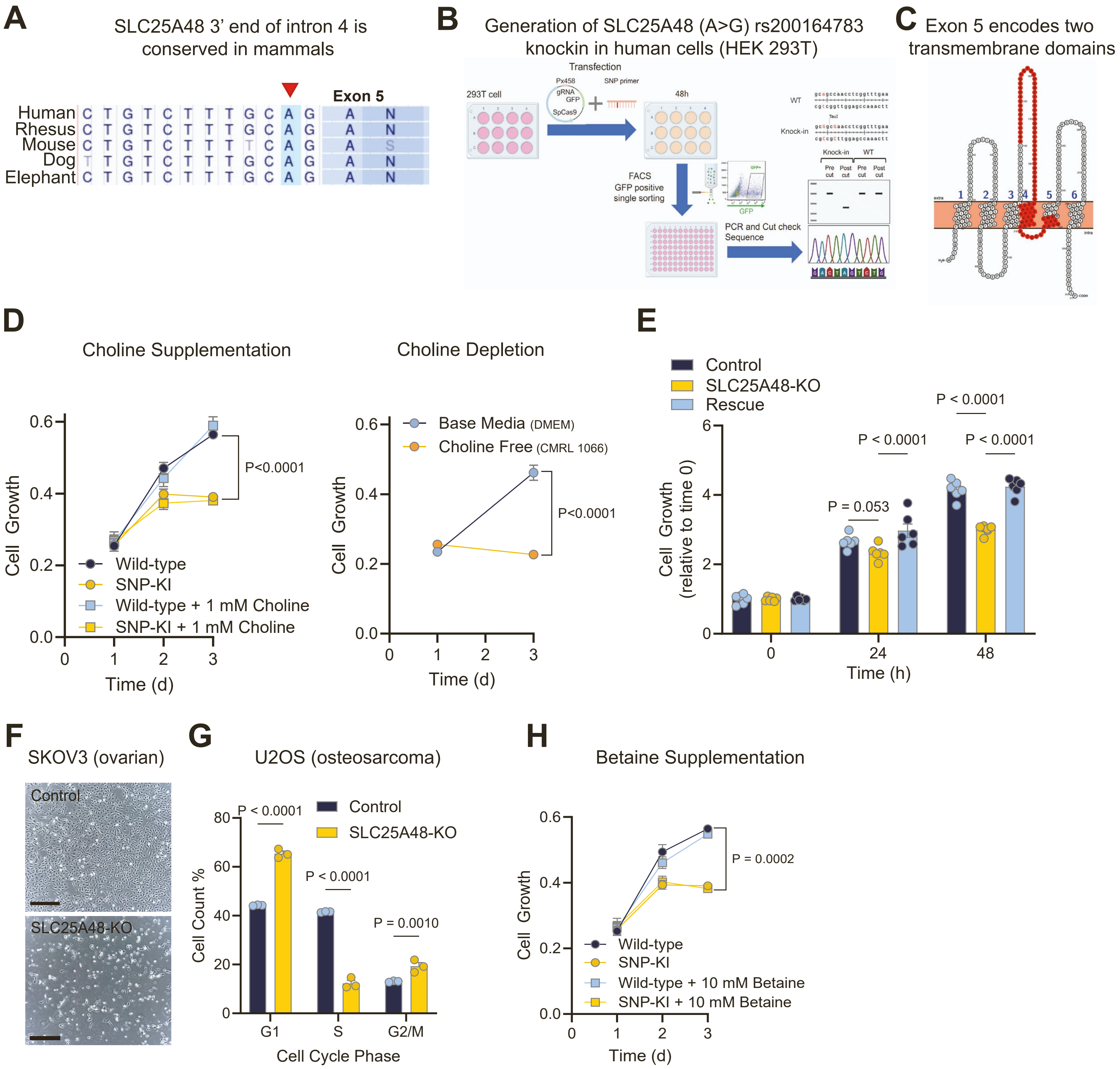

Figure S4. Analyses of SLC25A48-KI cells and SLC25A48 KO cells (Related to Figure 4)

A. Evolutionarily conserved sequences of the 3' end of Intron 4 splice recognition site (CAG) among indicated mammalian species.

B. Schematic for the generation of SLC25A48 (A>G) rs200164783 SNP-KI cells. HEK 293T cells were transfected with HDR primer of SNP mutation and Px458 plasmid containing GFP, Cas9, and sgRNA targeting SLC25A48. 48 hours after transfection, GFP-positive cells were sorted into single colonies. After cell expansion, single colony clones were screened for HDR transfection through restriction enzyme digestion. Selected clones were then sequenced to confirm specific DNA mutations. Wild-type control cells originated from the same parental cells as SNP-KI cells and lacked the expression of GFP, Cas9, and sgRNA.

C. The amino acid sequence of *SLC25A48* with Exon 5 is highlighted in red. Exon 5 encodes for two transmembrane domains. Exon 5 is not encoded in SNP-KI cells.

D. Cell proliferation over 3 days with choline supplementation (1 mM) or choline-free media. Cells were seeded on day 0 and given 24 hours to adhere before the addition of choline or choline-free media. Choline-free media (CRML 1066, no choline, 10% FBS, 1% P/S) was supplemented with glutamate, while the base medium was DMEM containing 10% FBS and 1% P/S. n = 4 per group for choline supplementation and 3 per group for choline depletion. Statistic: two-way repeated measures ANOVA with Dunnett's multiple comparisons test for choline supplementation and two-way repeated measures ANOVA with Šídák's multiple comparisons test for choline depletion.

E. Cell growth in HEK-293T cells is impaired by loss of SLC25A48. Control, SLC25A48-KO, and SLC25A48-rescued HEK 293T cells cell growth over 48 hours, relative to time 0 h. n = 6 per group. Statistic: two-way ANOVA with Tukey's multiple comparisons test. Bars represent mean with error shown as s.e.m., individual values presented.

F. Representative image of SKOV3 ovarian cancer cells transfected with control or SLC25A48-KO plasmids for 24 hours. Scale bar: 500 µm.

G. Cell cycle analysis of U2OS osteosarcoma cancer cells 16 hours after transfection with control or SLC25A48-KO plasmid. n= 3 per group. Statistic: two-way ANOVA with Šídák's multiple comparisons test. Bars represent mean with error shown as s.e.m., individual values presented.

H. Cell proliferation over 3 days with betaine supplementation (10 mM). Wild-type control and SNP-KI cells were seeded on day 0 and given 24 hours to adhere prior to betaine supplementation. n = 4 per group. Statistic: two-way repeated measures ANOVA with Dunnett's multiple comparisons test.

Table S1. Sequences of PCR Primers, related to STAR Methods

Mouse qPCR primers

Gene	Forward Primer	Reverse Primer
18S	AGTCCCTGCCCTTTGTACACA	CGATCCGAGGGCCTCACT
36B4	GGCCCTGCACTCTCGCTTTC	TGCCAGGACGCGCTTGT
Slc25a48	AGCAACTCCAATGGATGTCGT	CGCACAGCATTCACAGTGAT
Slc25a4	CAGGTGCGGAGTTGTCCC	TCCTCCAGCCTCTAAGGACC
Pgc1a	AGCCGTGACCACTGACAACGA	GCTGCATGGTTCTGAGTGCT
Adipoq	GCACTGGCAAGTTCTACTGCAA	GTAGGTGAAGAGAACGGCCTTGT
Fabp4	ACACCGAGATTTCCTTCAAACTG	CCATCTAGGGTTATGATGCTC
Cox4i	GCCTGATTGGCAAGAGAGCC	CAAGGGGTAGTCACGCCGAT
Cox7a	CAGCGTCATGGTCAGTCTGT	AGAAAACCGTGTGGCAGAGA
Cox8b	GAACCATGAAGCCAACGACT	GCGAAGTTCACAGTGGTTCC
Cox1	TAGCCCATGCAGGAGCATCA	TGGCTGGGGGTTTCATGTTGA
Cox3	CTTCACCATCCTCCAAGCTTCA	AGTCCATGGAATCCAGTAGCCAT
Apt6	TGGCATTAGCAGTCCGGCTT	ATGGTAGCTGTTGGTGGGCT
Atp8	TTCCCACTGGCACCTTCACC	TGTTGGGGTAATGAATGAGGCAA

Human qPCR primers

Gene	Forward Primer	Reverse Primer
18S	CGCCGCTAGAGGTGAAATTCT	CGAACCTCCGACTTTCGTTCT
SLC25A48	TTGGCTACGGAAACACCCTC	TGCGTGTTACTGAAGACCCC
SLC25A48 (Codon optimized cDNA)	GCCAGCATCGCCGTGTACAACT	GGGATGCCTCTGGCTCTCCACA
SLC25A48 (exon 4-5)	TCAAGATCCGGTTGCAGATGC	GTGCACTGGCCCCTGGTATG
