# Supplementary figures and images for: SLC25A48 controls mitochondrial choline import and metabolism
Source: Cell Metab. Author manuscript; Available in PMC 2025 Mar 29. (PMC11953726; doi:10.1016/j.cmet.2024.07.010)

**Figure S1B**

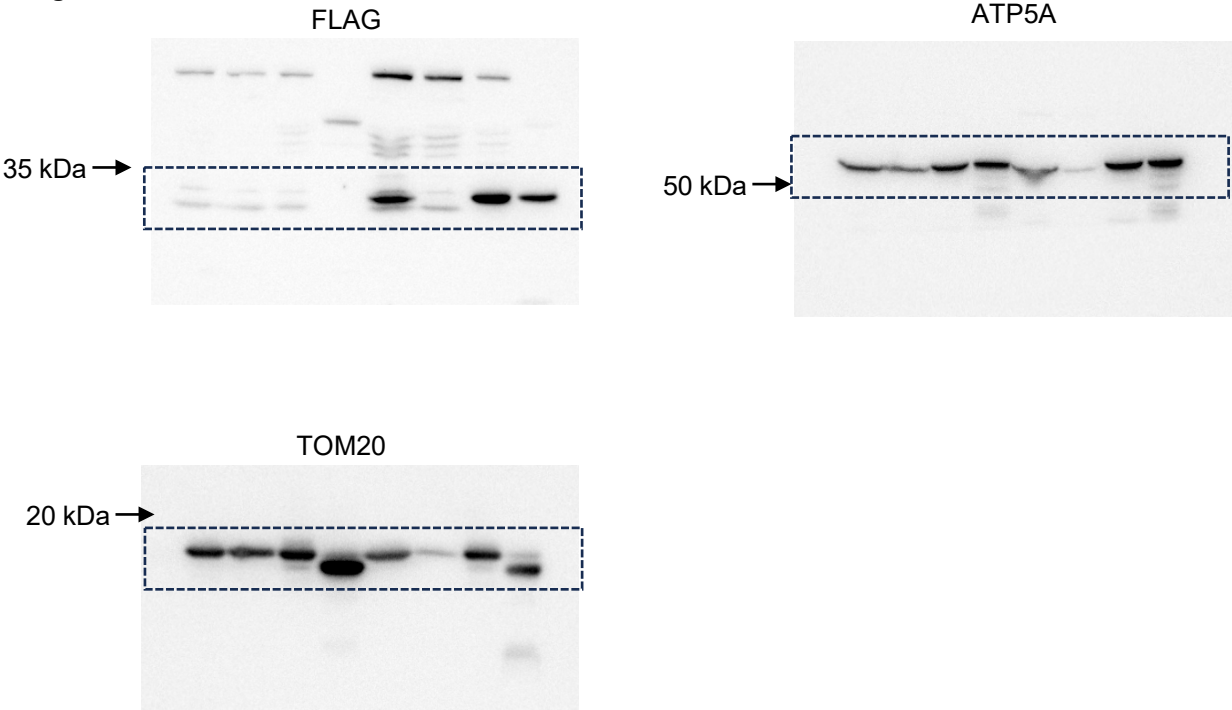

**Figure S1J**

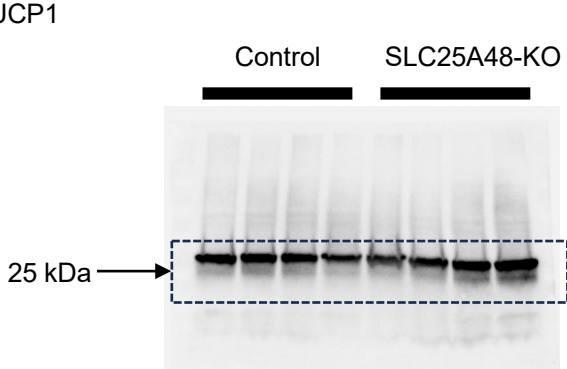

**Figure S1K**

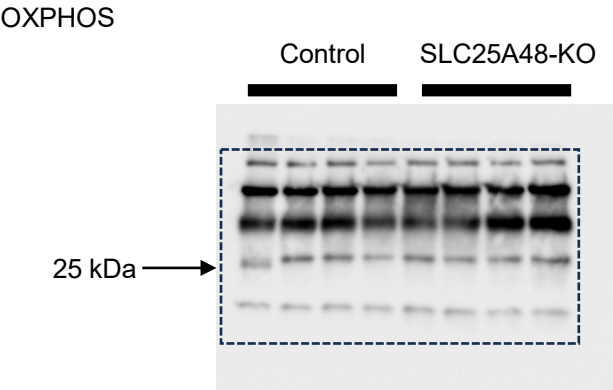

**Figure S2A**

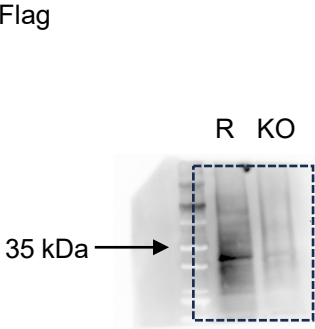

**Figure S2D**

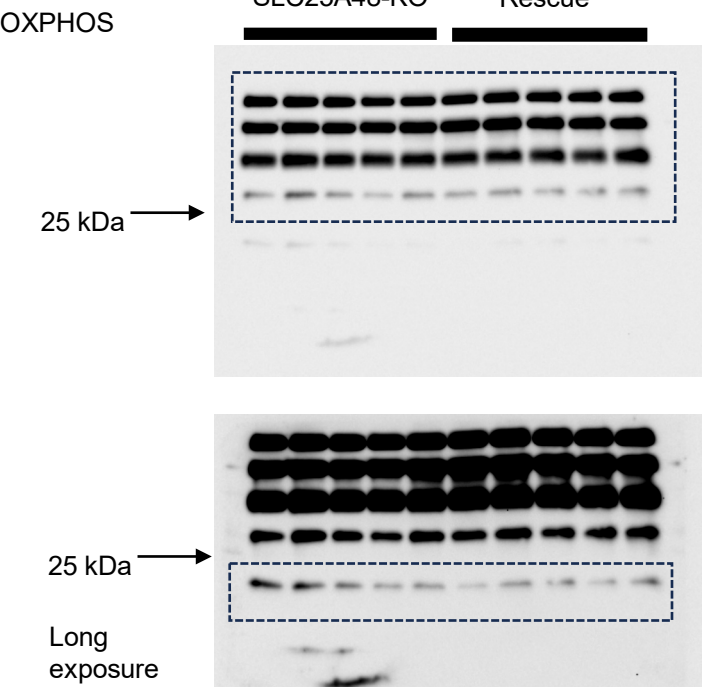

Supplement: source data S1 [file NIHMS2063854-supplement-source_data_S1.zip › Uncropped Western Blots.pdf]
